# Mantle cell lymphoma in the era of precision medicine-diagnosis, biomarkers and therapeutic agents

**DOI:** 10.18632/oncotarget.8961

**Published:** 2016-04-23

**Authors:** Arati A. Inamdar, Andre Goy, Nehad M. Ayoub, Christen Attia, Lucia Oton, Varun Taruvai, Mark Costales, Yu-Ting Lin, Andrew Pecora, Stephen K. Suh

**Affiliations:** ^1^ The Genomics and Biomarkers Program, The John Theurer Cancer Center, Hackensack University Medical Center, Hackensack, NJ, USA; ^2^ Clinical Divisions, John Theurer Cancer Center, Hackensack University Medical Center, Hackensack, NJ, USA; ^3^ Department of Clinical Pharmacy, Jordan University of Science and Technology, Irbid, Jordan

**Keywords:** biomarker, clinical trial, mantle cell lymphoma, personalized therapy, prognosis

## Abstract

Despite advances in the development of clinical agents for treating Mantle Cell Lymphoma (MCL), treatment of MCL remains a challenge due to complexity and frequent relapse associated with MCL. The incorporation of conventional and novel diagnostic approaches such as genomic sequencing have helped improve understanding of the pathogenesis of MCL, and have led to development of specific agents targeting signaling pathways that have recently been shown to be involved in MCL. In this review, we first provide a general overview of MCL and then discuss about the role of biomarkers in the pathogenesis, diagnosis, prognosis, and treatment for MCL. We attempt to discuss major biomarkers for MCL and highlight published and ongoing clinical trials in an effort to evaluate the dominant signaling pathways as drugable targets for treating MCL so as to determine the potential combination of drugs for both untreated and relapse/refractory cases. Our analysis indicates that incorporation of biomarkers is crucial for patient stratification and improve diagnosis and predictability of disease outcome thus help us in designing future precision therapies. The evidence indicates that a combination of conventional chemotherapeutic agents and novel drugs designed to target specific dysregulated signaling pathways can provide the effective therapeutic options for both untreated and relapse/refractory MCL.

## INTRODUCTION

## MANTLE CELL LYMPHOMA

**Figure 1:**
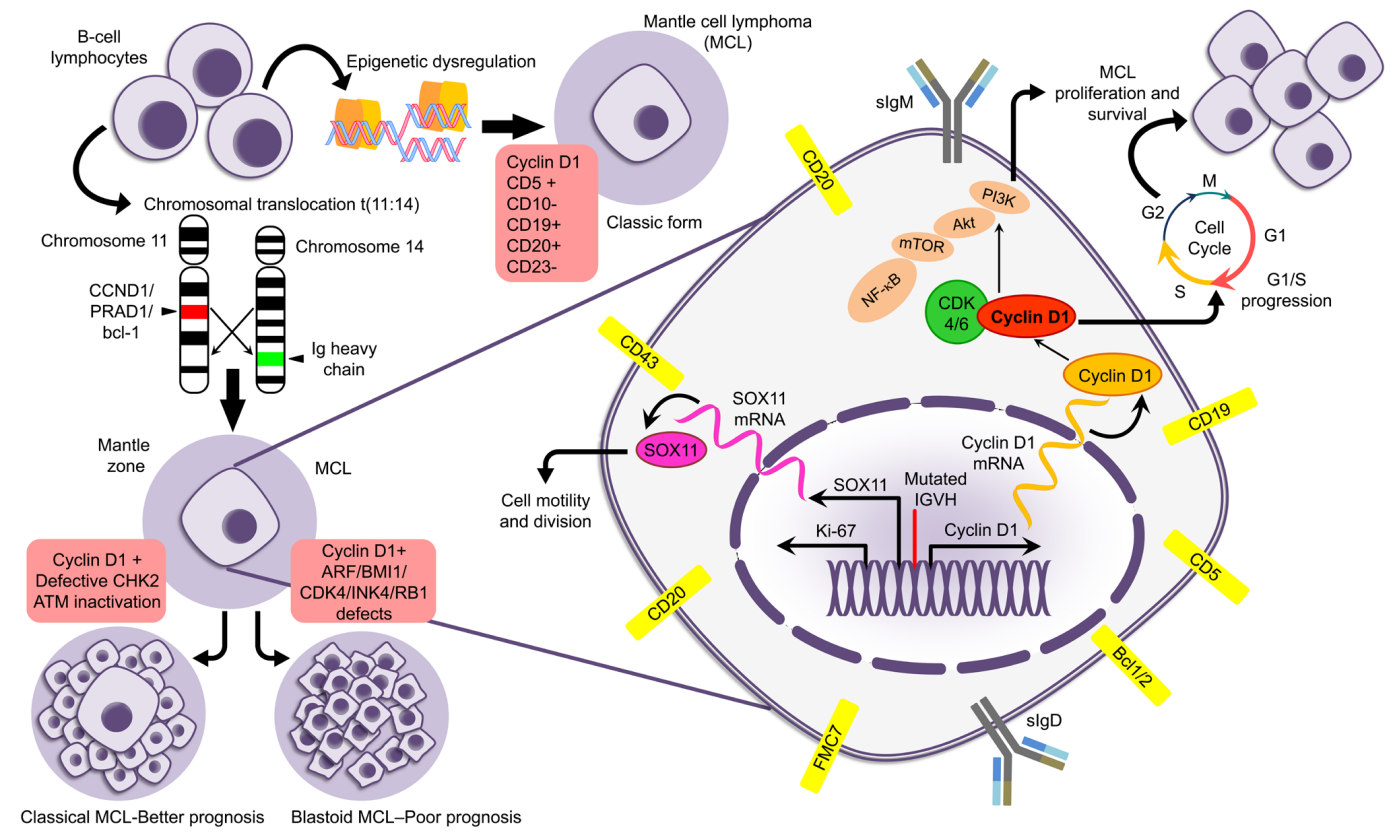
Figure describing the pathological and immunological details of MCL

Hematological malignancies are the seventh most common form of cancer and are the second leading cause of cancer-associated death (National Cancer Institute, USA). In the United States, tumors of B cell origin make up 85% to 90% of Non Hodgkin Lymphoma (NHL). Mantle Cell Lymphoma (MCL) is a subtype of B- cell derived NHL representing 5-6% of all NHL. MCL is a rare but aggressive B cell lymphoma that occurs more than four times as often in males as in females. The median age at diagnosis is about 60 years. MCL is derived from naïve, pre-germinal center cells of primary follicles or mantle regions of secondary follicles. MCL typically possesses the hallmark t(11;14)(q13;q32) chromosomal translocation, which causes overexpression of Cyclin D1, resulting in disordered progression of the cell cycle and aggressive lymphomagenesis. Although Cyclin D1-negative MCL cases have been reported, all such cases were positive for overexpression of cyclin D2 or D3 [1]. In addition, Sry-related high-mobility-group box (SOX11), which is expressed in nearly 90% of the cases, has been identified as diagnostic and prognostic biomarker of MCL [2]. The pathogenesis of MCL is complex and involves molecular alterations at various levels. Targeted genes and pathways include regulatory elements of the cell cycle machinery and senescence (ARF/BMI1/CDK4/INK4/RB1), DNA damage response pathways (ATM/CHK2/p53), and cell survival signals. Genes representing other signaling pathways including BTK, AKT, mTOR, WNT, NF-κB, TNF, and NOTCH have been found to play crucial roles in the pathogenesis of MCL (Figure 1) [3].

## CLINICAL CHARACTERISTICS OF MCL

### Clinical classification

MCL is classified into two forms: indolent and conventional. The indolent form is primarily characterized by a non-nodal leukemic presentation with bone marrow involvement and splenomegaly [4]. Most of these patients show normal performance status, normal serum LDH, and low MIPI score. Other characteristic features of indolent MCL include mild to moderate lymphocytosis, hypermutated IGVH genes, a non-complex karyotype, and absence of SOX11 expression [5] (Figure 1). Furthermore, cases of indolent MCL are associated with low Ki67 (≤10%) and kappa light chain expression as opposed to lambda light chain expression typically found in aggressive MCL. To date, markers which can confirm the diagnosis of indolent nature of MCL are lacking although SOX11 shows promise as one such biomarker [6].

### Clinical and pathological characteristics

At diagnosis, MCL patients typically present with generalized lymphadenopathy but with advanced disease, often at clinical stage III or IV. Systemic symptoms such as loss of appetite and weight loss, fever, night sweats, nausea and/or vomiting, indigestion, and abdominal pain or bloating are commonly reported. Gastrointestinal involvement is detected in 90% of cases of MCL, and 50% of patients present with blood and marrow involvement. MCL diagnosis is based on morphological resemblance of lymphoma cells to the mantle zone (MZ) B-lymphocyte lineage from which the tumor derives, and typically exhibit an MZ B cell phenotype: sIgM+, sIgD+, CD5+, CD20+, CD23−, FMC7+ [7]. Pathologically, MCL is further classified into two main subtypes: classic and blastoid, the blastoid form being associated with a more aggressive clinical course (Figure 1). Differential diagnosis for MCL includes Chronic Lymphocytic Leukemia (CLL) or Small Lymphocytic Lymphoma (SLL), Follicular Lymphoma, and marginal zone lymphoma [8]. CLL and SLL express sIgM, sIgD, CD19, and CD20, and have differential expression of T cell antigen CD5. However, MCL cells are positive for FMC7 and “typically” do not express CD23. They also exhibit greater staining intensity for B cell antigens and Igs. Like follicular lymphoma, MCL is positive for CD20 and Bcl-2, but in contrast to follicular lymphoma, MCL is negative for CD10, BCL-6, as well as CD23 [9]. Pathogenic mechanisms and phenotypical characteristics of MCL are illustrated in Figure 1.

### Laboratory studies

Blood and bone marrow examination, blood chemistry, lymph node biopsy, immunocytochemistry and flow cytometry, and cytogenetic studies aid in diagnosis of MCL. Usually complete blood count yields lymphocytosis along with anemia and cytopenia. Serum chemistry is significant for elevated LDH and elevated Beta-2-microglobulin. Wright-Giemsa staining is used for determining the presence of circulating lymphoma cells specific for MCL in peripheral blood. On microscopic analysis, the cancerous lymphocytes appear small to medium-sized with a condensed chromatin, scant cytoplasm, and small nucleoli. The morphological spectrum of leukemic MCL ranges from ‘small cells’ resembling chronic lymphocytic leukemia (CLL) or follicular lymphoma (FL) to ‘large cells’ mimicking prolymphocytic leukemia (PLL) or acute leukemia. Large cell morphology is associated with more frequent additional cytogenetic abnormalities as well as poorer outcome [10]. Bone marrow aspiration and biopsy slides show cellularity consistent with lymphoma and usually demonstrate a pattern of nodular, interstitial, paratrabecular, or diffuse involvement or, in some cases, a combination of these patterns [11]. MCL most commonly presents with a diffuse effacement of the lymph nodes; *in situ*, mantle-zone, nodular and diffuse, and diffuse patterns are also commonly seen [12]. Some of the cytologic variants seen on biopsy are marginal zone-like variant, small cell variant, blastoid cell variant, and pleomorphic variant; blastoid and pleomorphic variants are associated with a more aggressive clinical course.

Samples from bone marrow and lymph nodes are typically used for immunohistochemical and flow cytometry studies. Almost all cases of MCL show overexpression of Cyclin D1 messenger ribonucleic acid (mRNA). The classic immunophenotype is strongly positive for pan-B cell antigens CD5, CD19, CD43, weakly positive for FMC7, and negative for CD10, CD23, and Bcl-6. Variants of the classic immunophenotype have been identified flow cytometrically; these include BCL-1+/CD5− lymphoma with morphologic features consistent with MCL [13]. Gao et al. 2009 reported isolated cases that were CD 10+, CD 23+, and FMC7- as well as cases with variations in two antigens: CD5-/CD23+, CD10+/FMC7-, and CD23+/FMC7-. Multi-parameter flow cytometry is becoming an essential tool in characterization and diagnosis of variant forms of mature B cell lymphoproliferative neoplasms including mantle cell carcinoma [14, 15].

On cytogenetics studies, almost all MCL cases exhibit the reciprocal translocation t(11;14)(q13;q32) involving Cyclin D1 genes (CCND1, PRAD1, bcl-1) on chromosome 11 and the Ig heavy chain locus on chromosome 14. Conventional karyotyping detects this translocation in almost 65% of cases; however, the translocation is identified in up to 99% of cases by fluorescence *in situ* hybridization (FISH). A few cases of Cyclin D1-negative lymphoma with morphologic, pathologic, clinical, and molecular features typical of MCL have also been reported; these typically lack evidence of chromosomal translocations or genomic amplifications but possess high levels of cyclin D2 or D3 [1, 6]. Cytogenetic, comparative genomic hybridization (CGH), and expression profiling studies have been used to identify secondary genetic alterations that may be involved in the pathogenesis and progression of MCL [16, 17]. Numerous reports have confirmed that MCL carries the highest levels of genomic instability among the malignant lymphoid neoplasms.

## MOLECULAR TECHNIQUES FOR DIAGNOSIS OF MCL

Southern blot was utilized for detection of Ig and T cell Gene rearrangements associated with MCL and other B cell lymphomas until PCR replaced this laborious technique. PCR requires a small quantity of DNA (approximately 2 μg) and can be performed on paraffin tissue with minimal labor and short turnaround time. More recently, gene expression profiling (GEP) has provided a novel approach for diagnosis of hematological malignancies including lymphomas. [18]. Among various GEP platforms, RNA guided gene expression profiling of tumors measures the quantity of RNA transcripts which are labeled and hybridized on the array. Such GEP studies have been especially useful in detailed characterization and categorization of neoplasia into other subtypes and new variants of B cell derived lymphoma [19, 20]. When combined with RNA interference (RNAi), this technique has assisted in providing novel insights into signaling pathways associated with MCL [21]. cDNA microarray analysis allows detection of gain or loss in specific chromosomal regions, and has been used to describe the novel spectrum of somatic mutations of MCL [22]. Similarly, whole genome and whole exome sequencing allow measurement of the expression of thousands of genes simultaneously in a single experiment, and offer a powerful technology for the study of hematological malignancies including MCL [23]. Recently, whole transcriptome shotgun sequencing (RNAseq) identified a role of somatic mutations in NOTCH1 for the pathogenesis of MCL [24]. More recently, next generation sequencing (NGS) was found to be equally sensitivity as allele-specific oligonucleotides-PCR for immunoglobulin heavy-chain-gene-based minimal residual disease (MRD) detection, providing an effective tool for MRD monitoring [25]. The combination of tissue microarray and automated quantitative assessment of immunofluorescence (TMA-AQUA) as well as proteome- and microarray-based expression analysis of lymphoma cell lines *via* MS analysis (using MALDI-TOF) are capable of identifying large numbers of possible molecular targets relevant to MCL and other lymphomas [26–27]. All of these techniques have contributed to identification of underlying molecular mechanisms contributing to the pathogenesis and clinical progression of the disease, and have expanded our ability to predict clinical outcomes of lymphoma patients, as well as aided in identification of targets for novel therapeutic agents.

## ROLE OF TUMOR MICROENVIRONMENT IN THE PROGRESSION OF MCL

**Figure 2:**
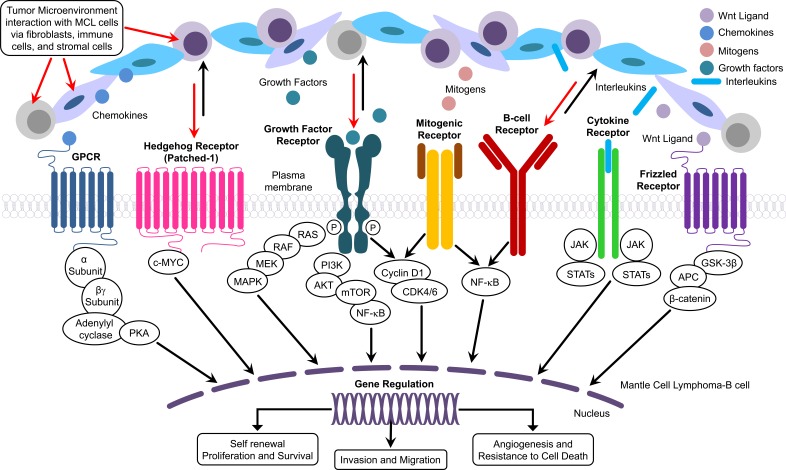
Figure describing the details of the interaction between components of TME and pathological pathways involved with MCL

Inflammation plays a crucial role in the initiation of cancer as well as in shaping the environment for survival of the tumor cells [28]. Local and systemic inflammatory responses induced by cytokines, chemokines, and small inflammatory proteins derived from tumor cells and/or host immune cells govern the major cross-talk between the tumor and host immune response. Therefore, it is imperative to identify and therapeutically target the cytokines, chemokines, and other crucial transcription factors that can disrupt the interaction between tumors and host immune response leading to aggressive forms of cancer with poor clinical outcome [29]. In fact, the interactions between cancer cells, immune cells, and tumor stromal cells in the tumor microenvironment (TME) are capable of modulating core features of malignancy including proliferation, self-renewal, homing, invasion, angiogenesis, immune evasion, and survival of cancer cells *via* immune cells, endothelial cells, and fibroblastic cells (Figure 2). Based on this theme, published reports have identified role of B cell-secreted cytokines, chemokines, and growth factors in dynamic and bidirectional signaling that promote the recruitment of adhesion molecules responsible for sustained signaling necessary for proliferation and survival of tumor cells [30, 31]. (Figure 2). The significance of the TME for MCL progression is illustrated by the observation that treatment of the Jeko-1 MCL cell line with anti-CXCR4 and anti-VLA-4 antibodies reduced cross talk between the tumor cells and stromal cells, decreased IL6 levels and phosphorylation of ERK1/2, AKT, and NF-κB, and increased the therapeutic sensitivity of Jeko-1 [32]. Similarly, an inhibitor of phosphatidylinositol-3-kinase (PI3K)/AKT/mammalian target of rapamycin (mTOR) pathway, NVP-BEZ235, inhibited TME-specific signaling pathways such as IL4 and IL6/STAT3, resulting in suppression of angiogenesis, migration, and tumor invasiveness in MCL cells [33]. Inhibition of IL-6 resulted in down-regulation of JAK2/STAT3 and PI3K/Akt pathways in MCL and abrogated IL-6-mediated protection of MCL cells [34]. Administration of an antibody against CCR7, a chemokine receptor known to cause lymphoid dissemination in many cancers, suppressed dissemination of MCL cells and reduced mortality in an animal model, suggesting the importance of TME in the pathogenesis and aggressiveness of MCL [35]. The dynamic interaction among the components of TME is integral to sustaining proliferation, angiogenesis, immune suppression, self-renewal, invasion, and migration, thus, promoting tumor progression and, ultimately, drug resistance (Figure 2).

## ROLE OF BIOMARKERS IN DETECTION, DIAGNOSIS, AND THERAPEUTIC RESPONSE OF MCL

Biomarkers have long been used to support diagnosis and to indicate prognosis of diseases. However, incorporation of molecular biomarkers as components of routine diagnostic panels for human diseases has gained greater importance in recent years. Biomarkers serve as a guide for risk assessment, screening, differential diagnosis, determination of prognosis, prediction of response to treatment, and for monitoring progression of disease. The main pathogenic events of cancer involve dysregulation of oncogenes, tumor suppressor genes, and DNA repair genes; thus, cancer biomarkers fall into various categories such as genetic biomarkers, cytogenetic biomarkers, epigenetic biomarkers, biomolecule/cancer antigen biomarkers, etc. The identification of unique biomarkers for diagnosis and prognosis of MCL has lagged compared to other cancers. Fortunately, blood/serum being a dynamic system harbors circulating components of tumors, including small molecules secreted by tumor cells, as well as other immune cell-secreted biomolecules, that reflect the underlying disease process. A comprehensive list of available biomarkers that inform diagnosis, prognosis, and functional drug interactions of MCL, along with their normal functions are presented in Table 1; some of these are discussed below.

**Table 1:** Table describing the key MCL biomarkers that inform diagnosis, prognosis, and functional drug interactions of MCL, along with their normal functions

Major Family	Biomarker	Class	Normal Function	Expression in MCL	Involved Pathway(s)	Interaction with MCL Drugs	Detection Technique	References
Cell-Cycle Regulation related markers	Cyclin D1	Protein	Regulator of CDK4 and CDK6	Up-Regulated	Cell Cycle	PD0332991 and others inhibit CDKs	IHC, PCR, FISH	215, 216
	Ki67	Protein	Necessary for cell proliferation and RNA transcription	Up-Regulated	Cell Cycle	COX 2 and CDK inhibitors decrease Ki67	IHC, PCR, FISH	134, 216, 217
	ATM	Protein Kinase	Induces cell cycle arrest, DNA repair, apoptosis	Down-Regulated	Homologous Recombination Repair and Cell Cycle regulation	KU 55933, KU-60019 and VE-821 inhibit ATM	IHC, PCR, FISH	216, 218
	CHK2	Protein	Tumor suppressor, regulates cell division	Down-Regulated	CDK-mediated phosphorylation and removal of Cdc6	AZD7762, CCT241533 hydrochloride and NSC 109555 ditosylate inhibit CHK2	IHC, PCR, FISH	216, 219
	CDK4	Gene	Catalyzes cell cycle G1 phase progression	Up-Regulated	Cell cycle regulation, especially CDK-mediated phosphorylation and removal of Cdc6	AZD-5438, Purvalanol A and Purvalanol B inhibit CDK's	IHC, PCR, FISH	216, 218
	RAN	Protein	Required for importing of proteins into nucleus as well as transporting RNA out of the nucleus. Involved in chromatin condensation and cell cycle regulation.	Up-Regulated	Regulation of nucleocytoplasmic transportation and mitotic spindle formation by forming CRM1/RAN GTP complex with Chromosomal region maintenance 1 (CRM1)	CRM1 inhibitors LMB (Leptomycin B and other LMB analogues) indirectly inhibit RAN	IHC, PCR, FISH	216, 220
	MYC	Protein	Controls DNA replication, causes B cell proliferation, regulates cell growth and apoptosis	Up-Regulated	ERK Signaling, PEDF Induced Signaling, ErbB signaling pathway	Covalent inhibitor of cyclin dependent kinase 7 (CDK7) inhibit MYC	IHC, PCR, FISH	216, 221
	TCL1A	Onco protein	T cell leukemia/lymphoma 1A promotes nuclear translocation of AKT1, enhances cell proliferation, promotes cell survival; interferes with NF-kB inhibitor IκB	Up-Regulated	P13K-Akt signaling where TLC1A acts as a co-activator of AKT	Rapamycin inhibits Tcl1/Akt/mTOR pathway	IHC, PCR, FISH	85, 216, 222
	p27	Gene	Inhibits CDK2 and CDK4, involved in G1 phase arrest	Down-Regulated	Cell cycle inhibitors and regulators	NSAIDS increase expression of p27	IHC, PCR, FISH	216, 223
	CHK1	Protein	CDK-mediated phosphorylation and removal of Cdc6, G2/M Checkpoints,	Down-Regulated	Coordinates response to DNA damage during cell cycle and regulates checkpoint in cell cycle	CHK1 inhibitor PF-00477736 works synergistically with Wee1 inhibitor (MK-1775) to induce apoptosis in MCL cell lines	IHC, PCR, FISH	216, 224
	BAFF-R	Gene	Enhances B-cell survival, regulates B-cell population	Up-Regulated	PEDF Induced Signaling, TNF, Akt and TGF-Beta Pathway	anti-BAFF antibody has therapeutic application against SLE but has not been tested against MCL	IHC, PCR, FISH	216, 225
Transcription Regulators	Sox11	Trans-cription factor	Plays role in determining cell fate	Up-Regulated	Notch, ERK Signaling	methylation of SOX11 promotor region reduces expression of SOX11	IHC, PCR, FISH	2, 48, 216
	STAT3	Protein	Transcription activator	Up-Regulated	growth factors, cytokines and interleukin signaling pathways	WP1066 with vorinostat has been tested against MCL. Other STAT-3 inhibitors are also available	IHC, PCR, FISH	216, 226, 227
	Nuclear Factor Kappa Beta	Protein	Controls DNA transcription, cytokine production, and cell survival	Up-Regulated	NFKB signaling pathway	proteasome inhibitor PS-341 or a specific pIκBα inhibitor, BAY 11-7082 have been used against MCL cell lines	IHC, PCR, FISH	216, 228
Tumor Suppression/Necr-osis related markers	Tp53	Gene	Codes for tumor suppressor protein p53	Up-Regulated	apoptosis and cell cycle related signaling pathways	Cyclic Pifithrin-alpha hydrobromide and CP 31398 dihydrochloride stabilizes p53	IHC, PCR, FISH	61, 62, 216
	INK4A	Protein Kinase	Tumor suppressor, slows down cell cycle	Mutated or Deleted	Cellular Senescence and Cell Cycle Regulation	HDAC inhibitors interact with INK4A to regulate the cell proliferation	IHC, PCR, FISH	133, 216
	RB1	Gene	Regulator of entry into cell division, tumor suppressor	Inactivated	CDK-mediated phosphorylation and removal of Cdc6, Cell Cycle Regulation	HDAC inhibitors increase RB1 expression in Myeloid-derived suppressor cells	IHC, PCR, FISH	216, 229
	MDM2	Protein	Mediates ubiquitination of p53	Up-Regulated	PI-3K cascade signaling, CDK-mediated phosphorylation and removal of Cdc6	AMG232, JNJ-26854165 and Nutlin-3 inhibits MDM2-p53 interaction	IHC, PCR, FISH	216, 230
	BCL-6	Gene	Transcriptional suppressor, suppresses genes related to differentiation, cell cycle, and apoptosis	Down-Regulated	IL4-mediated signaling events, B Cell Receptor, Fox O signaling pathways, Direct p53 effectors	Rituximab inhibits BCL66 Paraffin targets BCL-6	IHC, PCR, FISH	216, 231
	BCL-2	Protein	Regulator of apoptosis	Up-Regulated	PEDF Induced Signaling, TGF-Beta, ERK Signaling,	2,3-DCPE hydrochloride, ABT-199, ABT-263, ABT-737 and Apogossypolone inhibit BCL-2	IHC, PCR, FISH	216, 232
	BIRC3 (Baculoviral IAP Repeat Containing 3)	Protein	Anti-apoptotic protein regulates caspases and apoptosis, modulates inflammatory signaling and immunity	Mutated or Deleted	apoptosis and NFKB signaling pathway	Needs further research	IHC, PCR, FISH	216, 233
	PTEN (Phosphatase and tensin homolog)	Gene	Tumor suppressor	Mutated or Deleted	PI-3K cascade Signaling Pathway	several drugs under clinical investigation	IHC, PCR, FISH	216, 234
	TNFAIP/A20	Protein	A20-binding inhibitor of NF-κB	Mutated or Deleted	Akt, ERK, NFKB Signaling Pathways	Overexpression of ABINs inhibits NF-κB activation; needs further research	IHC, PCR, FISH	216, 235
	TNFRSF10B	Gene	Tumor Necrosis factor receptor, Transduces apoptosis signals,	Inactivated	Death Receptor, TNF, Akt and TGF-Beta signaling Pathway	Cisplatin is known to alter expression of TNFRSF10B	IHC, PCR, FISH	216, 236
Apoptosis-Related markers	repp86	Protein	Spindle assembly factor, required for assembly of microtubules during apoptosis,	Up-Regulated	Cell Cycle Checkpoint control, Aurora A, PLK1 signaling events, Cell cycle spindle assembly and chromosome separation	Not available	IHC, PCR, FISH	216, 237
	survivin	Protein	Inhibitor of Apoptosis, Regulates mitosis	Up-regulated	Cell cycle and IL-23 immune response pathway	Not available	IHC, PCR, FISH	216, 238
	FAF1	Protein	Mediates programmed cell death	Deleted	Apoptosis, TNF-alpha/NF-kB Signaling Pathway, Apoptosis and Autophagy	Not available	IHC, PCR, FISH	216, 239
RNA/DNA Repair Regulator related markers	POLE2	Protein	DNA binding, Protein Binding, DNA-directed DNA polymerase activity	Up-Regulated	Telomere C-Strand Synthesis, CDK-mediated phosphorylation and removal of Cdc6, DNA Repair, Mitotic G1-G1/S phases	Thioguanine Daunorubicin and Cytosine arabinoside targets POLE2	IHC, PCR, whole-genome and/or whole-exome sequencing	216, 236
	Topoisomerase Iiα	Protein	Cut strands of DNA to manage DNA tangles and supercoils, promotes chromosomal disentanglement	Up-Regulated	Cell Cycle, Chromatin regulation/acetylation	HU-331, ICRF-187/193, mitindomide and m-AMSA inhibits topoisomerase Iiα	IHC, PCR, whole-genome and/or whole-exome sequencing	216, 240
	IGF2BP2	Gene	RNA-binding factor, facilitates mRNA transport and storage	Up-Regulated	Binding of RNA by Insulin-like growth factor-2 mRNA binding proteins	Not available	IHC, PCR, whole-genome and/or whole-exome sequencing	216, 241

Please see Supplementary Table 1 access complete table.

### Cyclin D1

Cyclin D1 is encoded by the CCND1 gene which consists of 5 exons; alternative splicing generates Cyclin D1a and D1b isoforms. Cyclin D1a, a 30-kDa labile protein, forms a complex with the cyclin-dependent kinase CDK4 or CDK6 to promote cell-cycle entry. Cyclin D1 binds to the cell cycle kinase p34^cdc2^ and drives cells from G1 into S phase. Although Cyclin D1 is cell cycle-related protein it is generally absent in normal lymphoid tissue or B cell lines in the absence of the t(11;14)(q13;q32) translocation [36], which causes rearrangement of the bcl-1 proto-oncogene from chromosome 11 into the immunoglobulin heavy chain locus on chromosome 14, resulting in an overexpression of Cyclin D1 mRNA (also known as bcl-1 and PRAD1). Thus, abnormally expressed Cyclin D1 contributes to unopposed proliferation and downstream molecular events leading to higher and prolonged Cyclin D1 mRNA and protein expression, resulting in greater tumor proliferation and shorter survival of MCL patients [37]. Although t(11;14) translocations occur in almost 85% of MCL cases, non-MCL and other lymphoproliferative disorders may also exhibit t(11;14) translocations [38]. Expression of Cyclin D1 can be detected in formalin-fixed, paraffin-embedded (FFPE), tissue samples from MCL patients. Traditionally used methods include *in situ* hybridization, northern and southern blot, and cytogenetics; more recently used methods include immunohistochemical detection of Cyclin D1, PCR for bcl-1 rearrangement corresponding to t(11;14) translocation, and RT-PCR for Cyclin D1 mRNA expression. From the detection standpoint, direct visualization with DNA fiber fluorescence *in situ* hybridization provides 95-100% sensitivity; PCR provides the lowest sensitivity (30-50%) of these assays [39]. Because Cyclin D1 interacts with other signaling pathways including BCR/PI3K/AKT/mammalian target of rapamycin (mTOR), nuclear factor-κB (NF-κB), tumor necrosis factor (TNF), Hedgehog and WNT pathways, and the Bcl-2 family of apoptosis regulators, it is not surprising that Cyclin D1 is at the center of the pathogenesis of MCL, enhancing tumor proliferation, facilitating evasion of apoptosis, and reducing immune control [40].

### Cyclin D2/D3

Because mouse models have failed to demonstrate that overexpression of Cyclin D1 induces MCL, other genetic aberrations such as presence of a deregulated myc family gene with Cyclin D1 have been suggested to contribute to the pathogenesis of MCL [41]. Furthermore, recently reported Cyclin D1 negative MCL cases have exhibited overexpression of cyclin D2 and D3 [1]. Similar to Cyclin D1, cyclin D2 and D3 genes belong to the highly conserved cyclin family, and they function as regulators of CDK kinases. Cyclin D2 and D3 are expressed at high levels in some Cyclin D1-negative MCL cases. Cyclin D1-negative MCL is clinically and morphologically indistinguishable from conventional Cyclin D1-positive MCL. However, these cases have generally failed to exhibit any chromosomal translocations or gene amplifications involving cyclin D2 or D3 loci by FISH analysis. Deregulation of cyclin D2 or D3 expression in these MCL cases is mostly considered to be due to epigenetic mechanisms [1]. However, translocations of the cyclin D2 locus (chromosome 12p13) into either IGK or IGH loci have been reported as the initiating events for MCL pathogenesis [42, 43].

### B-cell specific biomarkers

The presence of specific cluster of differentiation (CD) antigens allows diagnosis of various types of leukemia and lymphoma. As a B cell lymphoma, MCL is characterized by the presence of characteristic B cell expression patterns with high (> 90%) expression of CD5, CD19, CD20, CD21, CD22, CD43, CD79a, sIg, and cIg, and low (< 10%) expression of CD10 and CD23. Variants such as CD5-negative [13], CD23-positive [44], and combinations of CD5-/CD23+, CD10+/FMC7-, and CD23+/FMC7- forms have been reported [7]. CD5-/CD23+ MCL is associated with a more indolent course and improved outcomes as opposed to ‘classic’ CD5+/CD23- MCL cases [44]. Usually in MCL, there is a lack of germinal center cell marker CD10, but in rare cases CD10+ cells exhibiting pleomorphic blastoid morphology are seen [45]. CD20 has been targeted in MCL with anti-CD20 antibodies (Rituximab), which causes death of tumor cells through several mechanisms including anti-proliferative effects and pro-apoptotic effects in B cells [46]. Similarly, anti-CD20 radio-immunotherapy has been used for treating lymphoma including MCL [46].

### Sox11

Apart from MCL, Sox11 is expressed in MCL, lymphoblastic lymphoma, some Burkitt lymphomas, and T-cell prolymphocytic leukemia, but is not known to be expressed in other lymphoid neoplasms. SOX11 is expressed in all aggressive forms of MCL (90-95%), and functions as diagnostic and prognostic marker for MCL [47, 48]. Detection of its expression is invaluable in identification of Cyclin D1-negative MCL [49]. Sox11 has been shown to control the oncogenic transcriptional network and effector genes responsible for B-cell transformation. The direct transcriptional targets of the SOX11 protein, DBN1, SETMAR, and HIG2, which are involved in cell motility and cell division, are highly correlative to the expression level of SOX11 [50]. Sox11-negative MCL cases mostly demonstrate an indolent course and a non-nodal presentation with splenomegaly and high WBC and lymphocyte counts when compared with SOX11-positive cases. Absence of Sox11 is not, however, an exclusive feature of indolent forms of MCL [51]. Sox11 is also an important minimal residual disease marker (MRD) used to monitor the clinical response to therapy and to predict relapse of MCL [52].

### IGVH

MCL is characterized by translocation of the immunoglobulin heavy chain locus, and somatic hypermutation of the rearranged variable region of the immunoglobulin heavy chain (IGVH) gene [53]. The sequencing of the IGVH region provides information on the clonal origin of chronic B cell malignancies including MCL. An absence of somatic mutations is consistent with origin from a pre-germinal center B cell; whereas, tumors that show somatic hypermutation arise either from germinal center cells or from post-germinal center memory cells. The “mutated” IGVH status is defined as < 98% homology to the germ-line. A few studies suggest that the lack of IGVH somatic mutations correlates with a more aggressive clinical course and possibly with shorter survival of MCL patients [54]. More recently, BCL1/IgH, and IgH-VDJ along with SOX11 and Cyclin D1 have been discussed as biomarkers for MRD-guided management of patients with mature B cell malignancies including MCL [55].

### Ki67

The Ki67 index, defined by the percentage of Ki67-positive lymphoma cells (determined by histopathological analysis) has been the single most powerful and established prognostic biomarker for overall survival of MCL patients. A high Ki67 index in the lymph node biopsy at the time of initial diagnosis, indicating a highly proliferative tumor, is predictive of a poor outcome [56]. The Ki67 index is employed in workup protocols for many tumors, especially breast cancer and other lymphoid neoplasms [56, 57]. Although, the Ki67 index shows a positive correlation with outcome and survival duration, no significant association has been demonstrated between the Ki67 index and clinicopathologic parameters including LDH levels, B-symptoms, tumor stage, extranodal involvement, and performance status [58]. The Ki67 index has become a valuable prognostic parameter for MCL patients treated with immunochemotherapy such as the combination of rituximab and first line therapy [59, 60].

### Other cytogenetic and epigenetic biomarkers

TP53, a tumor suppressor gene crucial for regulating cell division and preventing tumor formation, is commonly altered in human cancers including MCL. TP53 mutations have been found in 15-20% of MCL patients and are associated with significantly shorter overall survival (OS) and poor prognosis [61]. Interestingly, TP53 mutations have been found to be equally distributed in MCL regardless of SOX11 expression or IGVH mutations [22]. In multivariate analysis, TP53 was the only significant independent molecular marker that improved the prognostic value of MIPI [62]. Similarly, ATM (ataxia telangiectasia mutated) gene, which encodes for a serine-threonine kinase and belongs to the phosphatidylinositol-3 kinase (PI-3K) family, acts as an important tumor suppressor gene. MCL is characterized by inactivation of ATM gene (involving in class switch recombination in the Ig heavy chain locus), which may act synergistically with overexpressed Cyclin D1 to override cell-cycle checkpoint controls [63]. Consistent with this, inactivation of ATM is associated with frequent chromosomal imbalances [63]. Bea et al. 2013 performed whole-genome sequencing (WGS) of 29 MCL lines and whole-exome sequencing (WES) of 6 MCL cell lines; they identified recurrent mutations in ATM, WHSC1, MLL2, BIRC3, MEF2B, and TLR2 in addition to mutations in common MCL related genes, CCND1, SOX11, and TP53 [22]. Interestingly, ATM mutations were seen only in SOX11-positive tumors, whereas CCND1 mutations were preferentially detected in MCL with IGVH-mutations [22]. WHSC1, MLL2, and MEF2B all belong to a group of chromatin modifiers suggesting a role of epigenetic mechanisms in the pathogenesis of MCL. The potent oncogene c-myc is critically involved in the regulation of many growth-promoting signal transduction pathways and interacts with various down-stream signaling pathways; over-expression of c-myc is commonly seen in multiple human cancers including MCL [64]. C-myc is also associated with Bcl-2 translocations. Both Bcl-2 and Bcl-6 are anti-apoptotic proteins, and their translocation under the influence of altered c-myc lead to abnormal growth abilities of the affected cells and commonly confer an aggressive form of MCL [65]. Upregulation of BMI1 and downregulation of miR-16 in the MCL side population (SP) reduces apoptosis in these cells [66]. Downregulation of PAX5, a member of the paired box (PAX) family of transcription factors, correlates with an aggressive, highly drug-resistant, phenotype predictive of poor prognosis [67]. Overexpression of JARID1B and reduced histone H3K4 tri-methylation were associated with MCL and therefore, depletion of JARID1B caused up-regulation of histone acetylation of H3 and inactivation of Cyclin D1 leading to apoptosis of MCL cells [68].

### Other signaling pathway-related biomarkers

MCL pathogenesis also involves dysregulation in several signaling pathways [40]. Analysis of serum proteins provides an indirect but inexpensive means to monitor expression of genes encoding for cytokines and chemokines. The resulting expression profiles provide molecular signatures for mantle cell lymphoma. Cytokines and chemokines impart crucial cell signaling, pro-inflammatory, and immunomodulatory functions. Multiple studies have analyzed pre-treatment and post-treatment serum cytokines and chemokines of MCL patients with the aim of predicting prognosis and assessing the response to therapy. Recent report showed that the elevation of IL-12, IP-10, sIL-2Ra, MIG, IL-1RA, IL-8, MIP-1a, and MIP-1b in the serum of newly diagnosed MCL patients [69]. The elevated levels of sIL-2Ra, IL-8, MIG, MIP-1a, and MIP-1b were predictive of inferior event-free survival, and elevated sIL-2Ra, IL 8, and MIP-1b were predictive of a poor prognosis. Alteration in Toll like receptors (TLRs) is associated with B cell lymphomas including MCL [70]. Small RNAs including microRNA miR-127-3p, miR-615-3p, and miR-18b are associated with better overall survival of MCL patients, and provide prognostic markers that distinguish between indolent and aggressive forms [71–73]. Comprehensive information on these and other biomarkers, and notes on their roles in the pathogenesis of MCL, are compiled in Table 1. This table presents MCL related biomarkers from various cellular classes such as cell cycle regulators, transcription regulators, tumor suppression/necrosis, DNA/RNA repair regulators, immune/inflammatory and other signaling genes. The existence of these additional biomarkers, many of which have been identified during genome wide studies, illustrates the complexity and heterogeneity of this disease, and is consistent with reports indicating that MCL harbors the highest levels of genomic instability among malignant lymphoid neoplasms [22, 74].

## PROGNOSTIC MARKERS FOR MCL

Due to the paucity of early symptoms and biological heterogeneity, MCL is generally diagnosed at Stage III or IV. For staging of MCL, clinicians typically consider CBC, LDH, bone marrow aspirate and biopsy, while CT and PET to assess spread of the disease. PET scans were found to be superior to CT for predicting prognosis. A maximum standardized uptake value (SUVmax) of more than 5 from the area of most intense uptake correlated with inferior 5-year overall survival [75]. Mantle Cell Lymphoma International Prognostic Index (MIPI) is a widely used prognostic model which incorporates ECOG (Eastern Cooperative Oncology Group) performance status (ranging from 0-5), age, leukocyte count, lactic dehydrogenase, and tumor cell proliferation rate (Ki67 staining) to estimate prognosis. It allows stratification of MCL patients into low (44% of patients; median OS, not reached), medium/intermediate (35% of patients; median OS, 51 months) and high (21% of patients; median OS, 29 months) risk groups [76]. The initial prognostic significance of MIPI was dependent on the treatment regimen [77], but MIPI has recently been shown to be independent of treatment [78]. Other biomarkers and characteristic features of MCL that have shown prognostic significance for poor outcome include presence of blastoid or pleomorphic morphologic characteristics and high proliferation index at the time of diagnosis or after intense therapy [79]. In addition, high expression of eukaryotic initiation factor 4E, Myc and SOX11 overexpression, low TCL1 expression, TP53 alterations, lack of hypermutated heavy chain immunoglobulin variable regions, presence of higher absolute levels of monocytes, beta-2 microglobulin, monoclonal and polyclonal immunoglobulin free light chain, IL-2Rα, IL-8, and MIP-1β in the serum have been correlated with poor prognosis and inferior outcome [51, 69, 80–85]. CD3(+), CD8(+), and particularly CD4(+) T cells, if present in high numbers, are characteristic of the indolent form of MCL; a high CD4:CD8 ratio correlates independently with longer OS [86].

## THERAPEUTIC APPROACHES FOR MCL

### Early stage MCL

In 10-15% of patients, MCL is diagnosed at Stage I and II, also known as limited stage lymphoma. This subset is generally associated with an indolent course and better prognosis than patients diagnosed at advanced stages. The preferred modality of treatment for Stage I and II MCL, especially with non-bulky tumor load, is radiotherapy [6]. Multiple retrospective studies on small samples have indicated an affirmative effect of radiotherapy on various aspects of stage I and II MCL disease including progression free survival (PFS), OS, local disease control, and symptom relief [87]. A recently performed retrospective study on a large sample by Murthy et al. 2014 suggests that limited stage lymphoma patients receiving Radiation Therapy (RT) experienced improved OS; in addition, in multivariate analysis, administration of initial RT was associated with a significantly lower mortality rate [88].

Despite improved PFS and OS in patients receiving only radiotherapy, Stage I and II MCL patients often relapse within one year [89]. Therefore, a current recommendation includes shortened conventional chemotherapy induction followed by consolidating radiation for MCL patients carrying high tumor burden (> 5 cm are presented below) and/or having poor prognostic factors (blastoid features, high Ki67 indices (> 20-40%), high β_2_ microglobulin (> 3 mg/l), and central nervous system involvement [6, 90]. In such cases, systemic therapy as indicated for advanced stages (see below) would be appropriate; however, radiotherapy alone has been reported to be equally effective even in MCL patients with relapse or with bulky tumor and/or extra-nodal presentation [91, 92]. Based on clinical trials on indolent and untreated MCL (Table 2), intense chemo-immunotherapy in young patients appeared to induce complete remission but was associated with higher side effects [93]. The combination of Bendamustine and Rituximab has produced completion remission with minimal adverse effects (although prolonged Bendamustine treatment is associated with lymphocytopenia) [94, 95]. We would advocate for the clinical trials comparing traditional chemo-immunotherapy with Bendamustine/Rituximab in combination with Bruton's tyrosine kinase (BTK) inhibitors, as was recently examined in a Phase 1/1b trial for untreated and relapsed/refractory non-Hodgkin lymphoma [96] (Table 2).

**Table 2:** Table describing the details of the published clinical trials on the MCL drugs as single on in combination

Indolent and untreated MCL																					
Drug(s)	Drug mechanism	Condition	Toxicity	No. of Patients	Phase	CR	PR	OS	ORR	PFS	Year	References
R-CHOP or R-HyperCVAD W/O autologous stem cell transplantation	Chemo-immunotherapy	older patients with MCL	R-HyperCVAD > toxicity (infection, venous thrombosis, acute kidey injury, transfusion need) than R-CHOP	38	N/A	N/A	N/A	N/A	N/A	R-CHOP + ASCT: 3.2 Y R-HyperCVAD: 4 Y R-CHOP:1.6 Y	2015	[46]
Bendamustine-Rituximab (BR) Vs R-CHOP/R-CVP	B: mechlorethamine R:monoconal Ab R-CHOP/R-CVP: Chemoimmunotherapy	first-line treatment of indolent NHL or MCL	vomiting, drug-hypersensitivity reactions higher in patients treated with BR & peripheral neuropathy /paresthesia and alopecia higher in patients treated with R-CHOP/R-CVP	BR:224 R-CHOP/RCVP:223	3	BR:31% R-CHOP/R-CVP:25%	N/A	N/A	97% Vs. 91%	N/A	2014	[98]
Lenalidomide and Rituximab	Lenalidomide:immuno-modulatory agent, rituximab: anti-CD20 mAb	indolent B-cell or mantle cell lymphomas previously rituximab resistant	N/A	50, evaluable:43	2	N/A	N/A	N/A	L: 30.2%, L+R: 62.8%	22.2 months	2014	[21]
HyperCVAD MTX/Ara-C and rituximab	Chemo-immunotherapy	previously untreated MCL	One death secondary to myelodysplastic syndrome, grade 3 febrile neutropenia & grade 4 infection	49	2	47%	31%	6.8 Y	86%	4.8 Y	2013	[53]
CHOP &DHAP + rituximab followed by autologous stem cell transplantation	Chemo-immunotherapy	younger patients with MCL	No toxic death or unexpected toxicities	60	2	RCHOP: 12% R-DHAP:57%	N/A	Five Y 75%	(R)-CHOP:93% and R-DHAP:95%	83 months	2013	[47]
Bendamustine +Rituximab + Cytarabine (R-BAC)	Alkylating agent + anti-CD20 monoclonal antibody + anti-metabolite	untreated (A1) or relapsed or refractory (R/R) MCL (A2)	grades 3 to 4 thrombocytopenia (87% of patients) and febrile neutropenia occurred in 12%	40	2	A1:95% A2:70%	N/A	N/A	A1:100% A2: 80%	A1: 2-years was 95% ± 5%; 70% ± 10% for A2	2013	[118]
Bendamustine + Rituximab (A1) Vs. CHOP plus Rituximab(A2)	Alkylating agent + monoconal Ab Vs. Chemoimmunotherapy	first-line treatment for patients with indolent and MCL	haematological toxicity, infections, peripheral neuropathy, and stomatitis	A1:274(assessed: 261), A2: 275 (assessed:253)	3	N/A	N/A	N/A	N/A	A1:69.5 months, A2: 31.2 months	2012	[23]
rituximab, bortezomib, doxorubicin, dexamethasone and chlorambucil (RiPAD+C)	Chemoimmunotherapy+ Proteosome inhibitor	first line treatment for elderly MCL patients	(18%) experienced grade 3 neurotoxicity	39	2	0.51	N/A	N/A	79%	26 months	2012	[49]
R-CHOP followed by yttrium-90 (90Y) –ibritumomab tiuxetan	Chemoimmunotherapy followed by radioimmunotherapy	untrreated MCL pateients	no unexpected toxicities	56 patients were eligible	2	55%	N/A	N/A	82%	N/A	2012	[48]
Chlorambucil + Rituximab	Alkylating agent + monoconal Ab	Indolent MCL	No serious side effects	20	N/A	0.9	0.05	N/A	0.95	89% had 3 years PFS	2011	[252]
Bortezomib plus CHOP-Rituximab	proteasome inhibitor+ Chemoimmunotherapy	previously untreated DLBCL and MCL	neuropathy, grade 3/4 anemia, neutropenia & thrombocytopenia	76	1/2	DLBCL:86% MCL:72%	N/A	2Y DLBCL:70% 2Y MCL:86%	DLBCL:100% MCL:91%	2 Y DLBCL:64% 2 Y MCL: 44%	2011	[253]
R-HyperCVAD alternating with R-MA & without stem cell transplantation	Intense chemoimmunotherapy	untreated aggressive MCL	myelodysplasia/acute myelogenous leukemia, acute toxicity +8 deaths	97	2	87%	N/A	10Y:not-reached, 8Y:56%	97%	N/A	2010	[106]
(R)VAD+C	Chemoimmunotherapy	Newly Diagnosed MCL	very low hematologic toxicity	113	2	65% at end of treatment	N/A	N/A	73%	N/A	2010	[254]
CTAP alternating with VMAC	Intensive multiagent chemotherapeutic regimen	newly diagnosed MCL	well-tolerated with 4% treatment-related mortality (including HSCT)	25	2	N/A	N/A	5Y: 75%	74%	5 Y: 54%	2008	[255]
Fostamatinib	prodrug of the spleen tyrosine kinase (Syk) inhibitor R-406	MCl & other B cell lymphomas	neutropenia, diarrhea, and thrombocytopenia	60	1&2	NA	N/A	N/A	11% (1/9) for MCL	4.2 months	2007	[256]
Bendamustine, Vincristine + Prednisone (BOP) Vs. Cyclophosphamide, Vincristine + Prednisone (COP)	Chemotherapy	advanced indolent NHL and MCL	alopecia and leucopenia were more severe with COP.	164	3	BOP:22% COP:20%	N/A	N/A	BOP:61% COP:46%	N/A	2006	[257]
CHOP vs R-CHOP	chemotherapy Vs. chemoimmunotherapy	previously untreated patients with advanced-stage MCL	Acceptable, with no major differences between the two therapeutic groups.	122	3	7% Vs 34%	N/A	N/A	75% Vs. 94%	N/A	2005	[114]
R-HyperCVAD alternating with rituximab plus high-dose methotrexate and cytarabine.	chemoimmunotherapy	untreated aggressive Stage III/IV MCL	myelodysplasia/acute myelogenous leukemia & eight treatment-related deaths	97	2	87%	10%	3Y: 82%	97%	3 Y: 64%	2005	[258]
Intensive chemotherapy (A1) Vs. (ASCT) and rituximab(A2)	R: immunotherapy	stage III/IV MCL	febrile neutropenia, Mucositis, interstitial pneumonitis & herpes zoster	20	2	N/A	N/A	OS: 3Y A1: 65% A2:88%	N/A	A1: 29% A2:89%	2004	[259]
Hyper-CVAD and high-dose methotrexate/cytarabine followed by stem-cell transplantation	chemotherapy	aggressive Stage III/IV	Treatment-related death occurred in five patients	45	N/A	38%	55.50%	N/A	93.50%	3 Y	1998	[130]

Relapse/Refractory MCL												
Drug (s)	Drug mechanism	Condition	Toxicity	No. of Patients	Phase	CR	PR	OS	ORR	PFS	Year	References
Vorinostat + Rituximab	histone deacetylase inhibitor + monoconal Ab	newly diagnosed and relapsed /refractory indolent NHL	well tolerated,with the most common grade 3/4 adverse events being asymptomatic thrombosis, neutropenia, thrombocytopenia, lymphopenia, and fatigue	28;3 patients of MCL	2	0%	33%	N/A	33%	N/A	2015	[145]
Involved field radiotherapy	radiotherapy	MCL patients	N/A	25	N/A	68%	16%	N/A	84%	N/A	2015	[260]

A: arm, P: phase, Y: year, R-DexaBEAM: rituximab,examethasone, carmustine, etoposide, cytarabine and melphalan; HDT: high-dose therapy; aNHL: aggressive NHL; iNHL: indolent Lymphoma; N/A : Not available. Please see Supplementary Table 2 to access complete table.

### Late stage MCL

Stage III and IV MCL are known as aggressive/advanced MCL as they usually carry a high tumor burden with poor prognostic features. In these cases, treatment should be initiated after the diagnosis in both symptomatic and asymptomatic patients. Treatment is usually tailored individually based on patient's age, symptoms, and risk factors [97]. Young patients (≤ 65 yr, no major comorbidities) are generally given intensive immuno-chemotherapy with autologous stem cell transplantation along with maintenance therapy to achieve better PFS and OS while elederly patients usually receive therapies tailored towards the individual characteristics. The phases of treatment for treatment-naïve late stage MCL are presented below.

### Frontline/Firstline therapy

The ideal candidates for intensive strategies are patients aged 65 years or younger without significant comorbidities, while non-intensive strategies are reserved for elderly patients of more than 65 years or patients with significant comorbidities [98]. Several historical clinical trials have evaluated efficacy of various treatment regimens with respect to partial response (PR), complete response (CR), overall response rate (ORR), and OS; these have included CHOP, R-CHOP, Maxi-R-CHOP (R-CHOP followed by higher doses of cytarabine, followed by an autologous stem cell transplant); R-hyper-CVAD (Rituximab, cyclophosphamide, vincristine, doxorubicin, and dexamethasone alternating with high-dose cytarabine and methotrexate) with or without autologous stem cell transplantation; BR (Bendamustine and Rituximab); R-FCM (Rituximab, fludarabine, cyclophosphamide, and mitoxantrone); R-DHAP (Rituximab, dexamethasone, cytarabine and cisplatin); R-CVP (Rituximab, cyclophosphamide, vincristine, and prednisone); R-CBP (Rituximab, cyclophosphamide, bortezomib, and prednisone); R-VAD+C (Rituximab, vincristine, doxorubicin, dexamethasone, chlorambucil); and RiPAD+C (rituximab, bortezomib, doxorubicin, dexamethasone, and chlorambucil) (Table 2). An analysis data from the NCCN NHL outcomes database confirmed that there were no differences in OS or PFS among patients receiving R-HyperCVAD or RCHOP+HDT/ASCR [99]. Multiple studies have confirmed that treatment with R-CHOP alone is inferior to either of these regimens and VR-CAP (bortezomib, rituximab, cyclophosphamide, doxorubicin, and prednisone) regimen [100–101].

In elderly patients, treatment of MCL needs to be “personalized” based on biological age, comorbidities, and general performance status. A comprehensive geriatric assessment *via* questionnaire allows overall estimation of life expectancy and tolerance of treatment as well as identifies reversible health conditions that may interfere with cancer treatment. Based on these criteria, elderly patients have been divided into three categories: fit, compromised, and frail. The aim of treatment in fit patients is complete remission, and includes conventional frontline immunochemotherapy such as R-CHOP or BR or chlorambucil plus VADC (vincristine, doxorubicin, oral dexamethasone) or PEP-C (prednisone, etoposide, procarbazine, and cyclophosphamide) followed by maintenance with Rituximab alone (Table 2). In compromised patients, the aim of the treatment is to control disease progression while balancing the efficacy of treatment based toxicities due to underlying comorbidities or impaired organ function. Hence, dose-adapted chemotherapy such as BR or Rituximab and chlorambucil with or without novel therapeutic agents may be recommended. In frail patients, preservation of quality of life along with symptomatic control of the disease is the main therapeutic goal. Therefore, mild chemotherapy such as chlorambucil with rituximab, prednisone, etoposide, procarbazine, or cyclophosphamide in different combination are usually recommended without autologous cell transplantation (Table 2). Usually such patients benefit from combination R-CVP (Rituximab, cyclophosphamide, vincristine, and prednisone), or a newer regimen of R-CBP (Rituximab, cyclophosphamide, bortezomib, and prednisone) [6, 102]. In general, BR is becoming the treatment of choice over R-CHOP/R-CVP in older patients [94, 102], although addition of low dose cytarabine with BR has shown CR of 95% in untreated older patients [116]. An alternative combination of an initial regimen of cytarabine and rituximab alternating with R-CHOP and followed by cytarabine and fludarabine achieved 87% CR [117].

### Consolidation therapy

Consolidation therapy is relatively short term therapy mainly given after the frontline/induction therapy. Ionizing radiation, whether in the form of local radiation therapy, radioimmunotherapy, or total-body irradiation in preparation for autologous hematopoietic stem cell transplantation (ASCT) may be used for treating MCL in clinical practice. High dose chemotherapy with Carmustine/BCNU, etoposide, cytarabine, and melphalan (BEAM) followed by ASCT is also available. BEAM strategy has recently been refined by adding/replacing individual drugs from the BEAM group. Recent data showed that BeEAM (bendamustine, etoposide, cytarabine, melphalan) and Bortezomib-BEAM (V-BEAM) provide feasibility and efficacy similar to BEAM with slightly higher PFS and OS for treatment of MCL. Another newer strategy is to administer bortezomib in a combination referred to as VcR-CAP (bortezomib/Velcade, rituximab, cyclophosphamide, doxorubicin/Adriamycin, and prednisone) in previously untreated MCL patients [103]. Stem cell transplantation in MCL patients is increasingly being used as consolidation therapy following standard chemotherapy. However, this is somewhat controversial as some evidence suggests that chemotherapy without stem cell transplant is equally effective [104]. However, the inclusion of stem cell transplantation has shown promise in inducing long term disease free survival in MCL patients: a regimen of rituximab and ASCT produced durable remission in patients with first remission, and non-myeloablative allogeneic stem cell transplantation (NST) produced durable remission in patients with relapsed or refractory disease [105]. A group of patients who received frontline intensive induction immunochemotherapy (Maxi-CHOP alternating with high-dose cytarabine and BEAM/BEAC with ASCT) plus maintenance with Rituximab showed median overall survival and response duration longer than 10 years, and a median event-free survival of 7.4 years [106]. In a recent study, no significant differences were found in 5-year overall survival rates for autologous *vs*. reduced-intensity conditioning allogeneic hematopoietic stem cell transplantation in either early or late transplantation cohort [107].

Consolidation is often achieved with therapeutic agents in combination with ASCT or in place of ASCT, especially in elder compromised or frail patients. In a 10 year follow up of young patients treated with R-Hyper-CVAD alternating with Rituximab, methotrexate, and Cytarabine (R-M-A) without ASCT, it was reported that median OS had not been reached and that the median time to treatment failure (TTF) was 5.9 years [104]. High dose consolidation therapy such as with methotrexate/cytarabine or busulfan/melphalan before ASCT achieved high PFS in untreated aggressive MCL [108–109]. Other novel drugs such as temsirolimus, ibrutinib, and lenalidomide have been tested as agents for consolidative therapy [6]. The Eastern Cooperative Oncology Group (ECOG) trial E1499, which used 90Y-ibritumomab tiuxetan RIT consolidation after R-CHOP in newly diagnosed MCL, showed improved 5 year OS in both young and elderly patients [110].

### Maintenance therapy

Maintenance therapy is given after completion of induction therapy and is intended to achieve longer term remission. The primary criteria for maintenance agents include ease of administration, minimal toxicity, and efficacy of maintaining remission. Rituximab is the most commonly used agent for maintenance therapy for MCL of all stages; it was first approved for initial treatment of non-Hodgkin lymphoma in 1997. It is being used both as a single agent and in combination with newer drugs; the combinations have achieved greater CR, PFS, and improved OS in both young and older patients [111]. Interferon alpha as a maintenance therapy has been found to be inferior to ASCT or Rituximab and carries higher toxicity profile [112]. Newer drugs such as lenalidomide, which are primarily used in induction therapy, have also been evaluated for maintenance therapy as a single agent or in combination with other drugs, particularly rituximab [113]. Similarly, Ibrutinib, lenalidomine and bortezomib are being evaluated for maintenance in MCL and as a component of the frontline therapy *via* LyMa and MCL 0208 trials; Clinicaltrials.gov/NCT00921414, NCT02242097, NCT02354313 and EudraCT Number 2006-000386-11, and NCT00310037, respectively (Table 3; [114–115]).

**Table 3:** Status of active MCL clinical trials registered with NIH at clinicaltrials.gov

Drug	Drug mechanism	Condition	No. of Patients	Phase	Clinical trial number*(registered at clinicaltrials.gov)	First received year	Status
Ibrutinib maintenance	BTK Inhibitor	MCL	36	2	NCT02242097	2014	Recruiting
Lenalidomide maintenance versus observation	immuno-modulatory agent	Advanced MCL	300	3	NCT02354313	2014	Recruiting
ACP-196	BTK Inhibitor	relapsed or refractory MCL	Recruiting/estimated number:120	2	N+F2:I15CT02213926	2014	Recruiting
Bendamustine, Rituximab, Ibrutinib	Alkylating agent + monoconal Ab + BTK Inhibitor	Newly Diagnosed MCL	520	3	NCT01776840	2013	Active, not recruiting
GS-9973	Spleen tyrosine kinase (SYK) inhibitor	Relapsed/Refractory MCL, CLL, DLBCL & iNHL	280	2	NCT01799889	2013	Recruiting
GS-9973 + Idelalisib	Spleen tyrosine kinase (SYK) inhibitor & PI3Kδ inhibitor	Relapsed or Refractory Hematologic Malignancies (MCL,CLL, FL, DLBCL and iNHL)	200	2	NCT01796470	2013	Active, not recruiting
SGN-CD19A	anti-CD19 mAb linked to monomethyl auristatin F (MMAF), a cytotoxic agent	MCL	120	1	NCT01786135	2013	Recruiting
Ublituximab+Ibrutinib	Anti-CD20 monoclonal antibody+Bruton's Tyrosine Kinase (BTK) inhibitor	MCL,Chronic Lymphocytic Leukemia	60	2	NCT02013128	2013	Enrolling by invitation
AT7519M	CDK Inhibitor	Relapsed MCL	12	2	NCT01652144	2012	Completed; results are awaited
Carfilzomib, Lenalidomide, Rituximab	proteasome inhibitor + immuno-modulatory agent + monclonal Ab	Relapsed/Refractory MCL	68	1&2	NCT01729104	2012	Recruiting
IMMU-114	Humanized mAb against HLA-DR	Relapsed or Refractory NHL and CLL	50	1	NCT01728207	2012	Recruiting
CEP-9722 + Gemcitabine + Cisplatin	PARP inhibitor + antimetabolite deoxynucleoside analogue + inorganic platinum agent	MCL	24	1	NCT01345357	2011	Completed; results are awaited
CC-122 HCL	Pleiotropic Pathway Modulator	MCL	140	1	NCT01421524	2011	Recruiting
CDX-1127 (Varlilumab)	Monoclonal antibody targeting CD27	refractory or relapsed CD27 Expressing B-cell Malignancies and selected types of solid tumors	170	1	NCT01460134	2011	Recruiting
Ofatumumab + Bendamustine	Alkylating agent & CD20 antibody	MCL Ineligible for Autologous Stem Cell Transplant	76	2	NCT01437709	2011	Recruiting
Panobinostat + Bortezomib	histone deacetylase inhibitor (HDAC inhibitor) &Proteasome inhibitor	Relapsed and/or Refractory MCL	24	1	NCT01504776	2011	Completed; results are awaited
R-CHOP-14R-HIDAC followed by RIT/HDT/ASCR	Sequential Chemo-Radioimmunotherapy Followed by Autologous Transplantation	Untreated Advanced Stage MCL	96	1&2	NCT01484093	2011	Active, not recruiting
SAR245409+Rtuximab + Bendamustine	Phosphoinositide 3-kinase inhibitor (PI3K inhibitor)+monoclonal Ab+alkylating gent	Relapsed or Refractory MCL & other kinds of lymphoma	85	2	NCT01403636	2011	Completed; results are awaited
SNS01-T	Small inhibitory RNA molecule that blocks the expression of Factor 5A mRNA	MCL in Relapse and other B cell malignancies	15	1&2	NCT01435720	2011	Active, not recruiting
PD 0332991 + Bortezomib	Cyclin-dependent kinase 4 and 6 inhibitor & Proteasome inhibitor	Relapsed MCL	30	1	NCT01111188	2010	Unknown
Bendamustine + rituximab versus CHOP + rituximab	B: mechlorethamine R:monoconal Ab RCHOP:Chemoimmunotherapy	first-line treatment for patients with stage III or IV indolent or MCL	549	3	NCT00991211	2009	Completed; results are awaited
Tositumomab and Iodine I 131 Tositumomab followed by CHOP	iodine-131 labeled anti-CD20 murine IgG2a monoclonal antibody + chemotherapy	untreated MCL	25	2	NCT00992992	2009	Completed; results are awaited
Rituximab as maintenance	CD20 antibody	MCL	299	3	NCT00921414	2009	Completed; results are awaited
Vorinostat	histone deacetylase (HDAC) inhibitor	MCL and other B cell NHL	54	2	NCT00875056	2009	Active, not recruiting
Bortezomib as maintenance	Proteasome inhibitor	untreated MCL	151	2	NCT00310037	2008	Active, not recruiting
Clofarabine	Nucleoside analogue	Relapsed/Refractory NHL	25	1&2	NCT00644189	2008	Completed; results are awaited
Epratuzumab or rituximab	anti-CD22 + anti-CD20 monoclonal antibody	NHL patients receiving antibody treatment	500	Not available	NCT00398372	2006	Completed; results are awaited
RT-PEPC (Rituximab, Thalidomide, Prednisone, Etoposide, Procarbazine, Cyclophosphamide)	Monoclonal antibody, immunomodulatory, immunosuppressant drug, Topoisomerase inhibitor, alkylating agent	Relapsed Mantle Cell Lymphoma	46	2	NCT00151281	2005	Unknown
Cladribine	Antimetabolite	Mantle Cell Lymphoma	48	2	NCT00002879	1999	Completed; results are awaited

## TREATMENT OF RELAPSE/REFRACTORY MCL

Among NHL, MCL has a higher rate of relapse, possibly due to its heterogeneity and complexity. NCCN Guidelines provide choice of several therapeutics which may be used as single agents or as part of a combination therapy regimen for relapse/refractory MCL. High dose therapy along with stem cell transplantation is administered in patients with relapse/refractory MCL unless they have undergone high dose therapy and autologous stem cell transplant previously. Few studies have evaluated the benefits of salvage chemotherapy with traditional agents such as R-FCM (Rituximab, Fludarabine, cyclophosphamide, and mitoxantrone), R-GemOc (Rituximab, gemcitabine, oxaliplatin), R-DHAP, or BR [118–121]; novel agents have been preferred over these traditional chemotherapeutic agents for treatment of relapse/refractory MCL. Temsirolimus, bortezomib, lenalidomide, and ibrutinib are approved agents in the European Union. Initial clinical trials in which each of these four agents was used as a single agent against relapse and refractory MCL gave ORR as 33% for bortezomib, 28% for lenalidomide, 22% for temsirolimus, and 68% for ibrutinib [122–125]. Combinations of some of these novel agents with other novel or traditional agents have been explored (Table 3). Some recent clinical trials indicate that a newer proteasome inhibitor (Carfilzomib) can be combined with other agents including bendamustine, BEAM regimen followed with ASCT or CDK9 inhibitor although neurotoxicity may be a limiting factor [126–129]. Lenalidomide, an immunomodulatory agent, has shown promising results in MCL cases refractory to bortezomib, and in cases previously treated with other agents or combinations [124,130]. Ibrutinib, an inhibitor of Bruton's tyrosine kinase, has demonstrated outstanding efficacy in heavily pretreated relapse/refractory MCL with minimal side effects, high response rate, and high PFS; in combination with rituximab and bendamustine, OR of 94% and CR of 76% were achieved [96]. Current clinical trials have been designed with Ibrutinib, bortezomib, and other novel agents to determine the best possible combination for treating MCL (Table 2). In addition to therapeutic agents, radioimmunotherapy with 90Y-ibritumomab tiuxetan produced an ORR of 31%. In another study in 16 patients with history of relapse or refractoriness to treatment, high dose ^131^I-tositumomab followed by high doses of etoposide and cyclophosphamide produced a CR rate of 91%, an ORR of 100%, and PFS of 61% [131]. Based on published clinical trial results, single agents usually seem to fail but drug combinations have given somewhat better results especially for relapse/refractory MCL (Table 2). Trials that have included Bendamustine in the regimen seem to induce complete remission, but more phase 3 clinical trials are required to demonstrate feasibility of the combination of bendamustine/rituximab with BTK inhibitors to achieve higher remission rates [96, 126, 132].

Major clinical trials and their outcomes for indolent, untreated and refractory/relapse MCL are described in chronological order in Table 2. The data demonstrate how clinical trials have been useful in refining treatment strategies over decades. Newer clinical trials incorporating combination chemotherapeutic agents with novel MCL drugs are described in Table 3. These newer clinical trials (Table 3) include both ongoing trials and trials in the recruitment stage; these trials will help us to evaluate putative beneficial roles of novel therapeutic agents especially during treatment of first-time/untreated MCL. We anticipate that the results will provide avenues for reducing side effects of chemotherapeutic agents and minimize the relapse rates.

## NOVEL DRUGS FOR MCL BASED ON SIGNALING PATHWAYS

**Figure 3:**
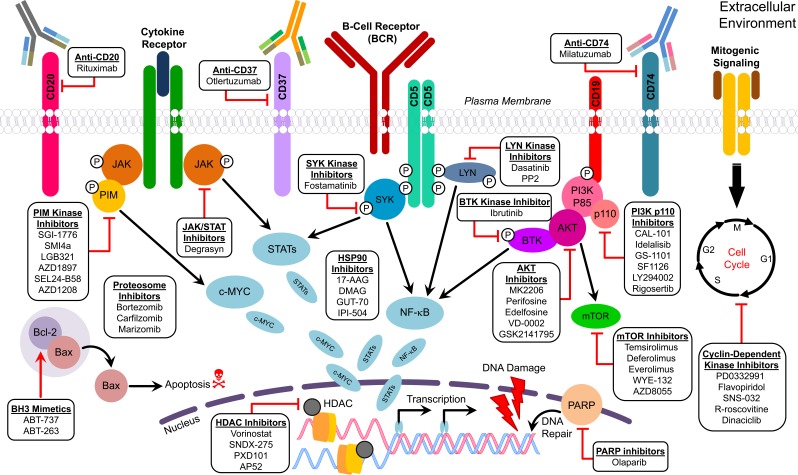
Figure describing the molecular targets of therapeutic agents used for MCL

Several research groups have taken a novel approach aimed at targeting the molecular mechanisms involved in pathogenesis of MCL. Many of these therapeutic agents have shown promise in preclinical studies and clinical trials. The properties of these chemotherapeutics and novel drugs currently in use or being developed for MCL, which may help to elucidate the therapeutic effect of these drugs against MCL, are detailed in Table 4. Cellular proteins and signaling pathways that could be targeted with novel inhibitors for treatment of MCL are described below (Figure 3).

**Table 4:** The pharmacological details of the drugs currently in use or being developed for MCL. Please see Supplementary Table 4 to access complete table

FRONTLINE DRUGS								
	Generic name Trade/Pipeline Name(s)	Company/FDA Approval year	Chemical structure	Drug Class	Status/Route	Target/Mechanism of action	Toxicity	References
1	**Chlorambucil** Leukeran Ambochlorin Amboclorin Linfozilin	ASPEN GLOBAL INC, 1957	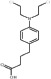	Alkylating agent	Prescription/Oral	**Target:** DNA synthesis. **Mechanism of action:** Binds DNA preventing – DNA synthesis. – RNA transcription. – Mutagenic effect. Induces cellular apoptosis: – Accumulation of cytosolic p53. – Activation of Bax.	**Hematologic:** Bone marrow suppression, anemia, leukopenia, neutropenia, thrombocytopenia, pancytopenia. **Gastrointestinal:** Gastrointestinal disturbances **Dermatologic:** urticaria, angioneurotic edema, skin hypersensitivity. **Other:** pulmonary fibrosis, hepatotoxicity, fever, peripheral neuropathy, interstitial pneumonia, sterile cystitis, infertility, leukemia, secondary malignancies.	[277]
2	**Lenalidomide** Revlimid	CELGENE, 2005	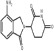	Immunomodulatory Agent	Prescription/Oral	**Target:** TNF-alpha inhibitor. **Mechanism of action:** Increases tumor cell apoptosis: – Enhancing IL-10 Induces G0-G1 cell cycle arrest: – CDK2 inhibition. T- cells activated via B7 pathway: – Tyrosine phosphorylation of CD28.	**Hematologic:** Neutropenia, thrombocytopenia, anemia. **Other:** pneumonia, fatigue.	[278]
3	**Ibrutinib** Imbruvica	PHARMACYCLICS INC, 2014	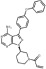	Antineoplastic Agent/Bruton tyrosine kinase inhibitor	Prescription/Oral	**Target:** Bruton's tyrosine kinase inhibitor **Mechanism of action:** Inhibits BTK and activates pathways necessary for B-cell trafficking, chemotaxis, and adhesion. Promotes cancer cell apoptosis, inhibits cell proliferation.	**Gastrointestinal:** Diarrhea, nausea, peripheral edema, constipation, vomiting, loss of appetite. **Other:** fatigue, dyspnea, upper respiratory tract infection.	[279]
4	**Methotrexate** TrexallRasuvoOtrexupMexate	BARR, 2001MEDAC PHARMA INC, 2014ANTARES PHARMA INC, 2013BRISTOL, 1979	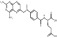	Antineoplastic Agent, Antipsoriatic, Antirheumatic, Cytotoxic	Prescription/Injection, Sub-cuteneous	**Target:** Dihydrofolate reductase inhibitor **Mechanism of action:** Inhibits folic acid reductase, hinders DNA synthesis and prevents cellular replication.	**Hematologic:** aplastic anemia, pancytopenia, leukopenia, neutropenia. **Other:** lymphadenopathy, drowsiness, blurred vision, cognitive dysfunction, moodiness, tinnitus, mucositis.	[280]
5	**Fludarabine** FludaraOforta	GENZYME CORP, 1991SANOFI AVENTIS US, 2008	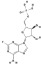	Purine Nucleotides, Antimetabolites Antineoplastic Agents	Prescription/Intravenous, Oral	**Target:** DNA synthesis inhibitor **Mechanism of action:** Inhibition of ribonucleotide reductase, incorporation into DNA: repression of DNA polymerization Inhibition of DNA ligase and DNA primase.	**Hematologic:** neutropenia, thrombocytopenia, anemia, lymphocytopenia. **Gastrointestinal:** nausea, vomiting. **Other:** infection (typically respiratory tract), fever, elevation of liver enzymes.	[281]
6	**Ibritumomab tiuxetan** Zevalin	SPECTRUM PHARMS, 2002	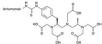	Carboxylic Acids and Derivatives	Prescription/Intravenous	**Target:** CD20 **Mechanism of action:** Ibritumomab tiuxetan binds to the CD20 antigen on Bcells. The CD20 antigen is not shed from the cell surface and does not internalize upon antibody binding. The chelate tiuxetan binds Y-90, is covalently linked to ibritumomab. The beta emission from Y-90 induces cellular damage: formation of free radicals in the targeted and neighboring cells.	**Hematologic:** Haemopoetic, myelosuppression	[282]
7	**Temsirolimus** Torisel	PF PRISM CV, 2007	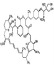	mTOR inhibitor, Antineoplastic agent	Prescription/Intravenous	**Target:** mammalian target of rapamycin (mTOR) inhibitor **Mechanism of action:** Binds to FKBP-12, and the protein-drug complex inhibits mTOR. Inhibition of mTOR activity results in a G1 growth arrest in treated tumor cells.	**Hematologic:** thrombocytopenia, asthenia, anemia. **Gastrointestinal:** diarrhea. **Other:** fever.	[283]
8	**Cyclo-phosphamide** CytoxanNeosar	BAXTER HLTHCARE, 1959TEVA PARENTERAL, 1993BEDFORD, 1982	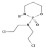	Alkylating agents	Prescription/Oral, intravenous	**Target:** DNA **Mechanism of action:** Forms DNA crosslinks both between and within DNA strands at guanine N-7 positions leading to cell apoptosis.	**Dermatologic:** alopecia. **Other:** haemorrhagic cystitis, immunosuppression (when not desired).	[284]
9	**Vincristine** Marqibo Kit Oncovin Vincrex Vincasar PFS Leurocristine	TALON THERAP, 2012LILLY, 1963BRISTOL MYERS SQUIBB, 1988TEVA PARENTERAL, 1987	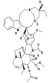	Antineoplastic Agents, Phytogenic	Prescription/Intravenous	**Target:** beta-tubulin, tubulin polymerization inhibitor **Mechanism of action:** Inhibits cell division during early mitosis. Binds to tubulin monomers preventing the formation of spindle microtubules. Stops the separation of the duplicated chromosomes and prevents cell division.	**Other:** acute uric acid nephropathy, acute shortness of breath and severe bronchospasm.	[285]
10	**Doxorubicin** Adriamycin PFS Rubex Doxil	PHARMACIA AND UPJOHN, 1987BRISTOL MYERS SQUIBB, 1989JANSSEN RES AND DEV, 1995	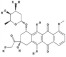	Antibiotics, Antineoplastic, Anthracyclines.	Prescription/Intravevous	**Target:** topoisomerase II inhibitor **Mechanism of action:** Doxorubicin is oxidized to an unstable metabolite and converted back to doxorubicin in a process that releases reactive oxygen species, this leads to lipid peroxidation and membrane damage, DNA damage, oxidative stress, and triggers apoptotic pathways of cell death. Alternatively, doxorubicin can enter the nucleus and poison topoisomerase-II, also resulting in DNA damage and cell death.	**Other:** acute and chronic cardiotoxicity/cardiomyopathy.	[286]
11	**Dexamethasone** Ciprodex Decaderm Decadron Dexamethasone Hexadrol Maxidex Ozurdex Tobradex ST	ALCON PHARMS LTD, 2003 MERCK, N/AMERCK, 1958 ECR, 2008 SANDOZ, 1971 WATSON LABS, 1973 PVT FORM, 1973 PHOENIX LABS NY, 1974 MUTUAL PHARM, 1974 ROXANE, 1975 STI PHARMA LLC, 1976 PAR PHARM, 1983 LYNE, 2011 VINTAGE PHARMS, 2011 ORGANON USA INC, 1962 ALCON, 1962 ALLERGAN, 2009 ALCON PHARMS LTD, 2009	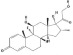	Glucocorticoid, Corticosteroid	Prescription/intravenous, intramuscular, intra-articular, intralesional and soft tissue injection	**Target:** glucocorticoid receptor agonist **Mechanism of action:** Inhibition of inflammatory agents in the body, implicated in the development or growth of some cancers.	**Gastrointestinal:** gastritis. **Other:** cushingoid facies, obesity, glucose intolerance, hypertension, psychologic alterations, infections, myositis and myopathy, avascular necrosis of bone.	[287]
12	**Cytarabine** Cytosar-UDepocyt	TEVA PARENTERAL, 1969 EUROHLTH INTL SARL, 1989 HOSPIRA, 1990 FRESENIUS KABI USA, 2004 MYLAN LABS LTD, 2011 TEVA PHARMS USA, 1998 PACIRA PHARMS INC, 1999	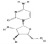	Antimetabolite antineoplastic agent, Nucleoside Metabolic Inhibitor	Prescription/Intravenous, Intrathecal, or Subcutaneous	**Target:** DNA polymerase inhibitor **Mechanism of action:** Damages the S phase of cell cycle. Inhibits both DNA and RNA polymerases and nucleotide reductase enzymes needed for DNA synthesis.	**Hematologic:** anemia, leukopenia, thrombocytopenia, megaloblastosis. **Other:** infection, Ara-C syndrome.	[288]
**RELAPSED DRUGS**								
13	**Bendamustine** Treanda Ribomustin Levact Cytostasan SDX-105	CEPHALON, 2008	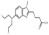	Alkylating/Antineoplastic Agent	Prescription/intravenous injection	**Target: DNA** **Mechanism of action:** Activation of DNA damage stress response and apoptosis induced by p21 (Cip1/Waf1) and Noxa, inhibition of mitotic checkpoints, and induction of mitotic catastrophe	**Hematologic:** febrile neutropenia. **Other:** fatigue, pneumonia, hypokalemia dehydration, pyrexia.	[289]

### CDK inhibitors

MCL involves aberrant expression of Cyclin D1 which causes dysregulation of regulators of the cell-cycle including CDK4. CDK4 is especially important for regulating the G1-S phase transition; its activity is negatively regulated by CDK inhibitors p16INK4A, p27Kip1, and p21Waf1/Cip1. The CDK4/6 inhibitor, PD0332991 has shown promise in both preclinical and clinical studies for treating MCL [133]. In a pilot study on relapse/refractory MCL patients, activity of PD0332991 was monitored *via* 3-deoxy-3[18F]-fluorothymidine (FLT) and 2-deoxy-2-[18F] fluoro-D-glucose (FDG) positron emission tomography/computed tomography (PET/CT) imaging as well as with Ki67 staining of the biopsy samples. There was > 70% reduction in summed FLT SUV (max), > 90% reduction of expression of phospho-Rb, and ≥ 87.5% reduction of expression of Ki67, although no long term changes in disease progression were noted [134]. Flavopiridol, a broad cyclin-dependent kinase inhibitor has been shown to induce apoptosis and to down regulate cell cycle proteins and anti-apoptotic proteins in *in vitro* models [135]. Flavopiridol showed limited utility against MCL as a single agent, but in combination with fludarabine and rituximab it showed median PFS of 21.9 months and 70% CR [136]. The effect of Flavopiridol can also be potentiated by combination with agents targeting other molecular pathways, including Hsp90 inhibitor 17-AAG [137]. In phase 1 clinical trials, flavopiridol in combination with the proteasome inhibitor, bortezomib, showed efficacy for treatment of MCL patients with relapsed/refractory status [138]. Other CDK inhibitors such as SNS-032, an inhibitor of cdk2, 7, and 9 act by inhibiting RNA pol II phosphorylation and reducing Mcl-1 levels [139]. From the therapeutic standpoint, small-molecule CDK inhibitors such as CYC202 (Seliciclib, R-roscovitine), a purine analogue and a selective inhibitor of cdk2-cyclin E, cdk7-cyclin H, and cdk9-cyclin T has been shown to induce increased expression of apoptosis regulatory genes and decreased Cyclin D1 in MCL cell lines REC, Granta-519, JeKo-1, and NCEB-1 [140]. Similarly, other cyclin-dependent kinase inhibitors including P276-00, SNS-032 and flavopiridol have shown clinical activity against MCL [141]. SCH727965 (dinaciclib), a novel inhibitor of CDK1, 2, 5, and 9, showed superior activity and an improved therapeutic index compared with flavopiridol in laboratory models of solid and hematologic malignancies [142]. In *in vitro* and *in vivo* studies using silvestrol, Alinari et al. 2012 found reductions of phosphorylated Rb, E2F1 protein, and E2F1 target transcription as well as mitochondrial depolarization and caspase-dependent apoptosis [143]. Chiron and colleagues showed that inhibition of CDK4 with PD 0332991 (palbociclib) sensitizes ibrutinib-resistant lymphoma cells to ibrutinib in the absence of BTK mutations; however, use of PI3K inhibitors was more effective for treating cells carrying a BTK mutation (C481S) [144]. Similarly, in a recent trial with vorinostat and Rituximab, 33% of patients (*n* = 3) achieved PR and ORR [145]. Additional clinical trials have been designed to evaluate the efficiency of CDK inhibitor alone and in combination for MCL (Table 3).

### BCR inhibitors

B cell receptor (BCR)-mediated signaling pathways are linked with expression adaptor molecules (e.g., GAB1, BLNK, GRB2, CARD11), activities of kinases (e.g., LYN, SYK, PI3K), and phosphatases (e.g., SHIP-1, SHP-1, PTEN) leading to inhibition of NF-κB, ERK, mTOR, and GSK3 pathways. Since MCL is a B cell derived lymphoma, the BCR and its associated signaling pathways are central players in supporting the B cell microenvironment-based B cell homing, survival and drug resistance [146]. Therefore, suppression of BCR signaling pathways *via* therapeutic agents which can block the upstream or downstream signaling molecules should lead to apoptosis of MCL cells. Furthermore, inhibition of BCR pathways also interferes with BCR-regulated interactions with the TME, which leads to disruption of secretion of pro-survival chemokines, increased release of lymphoma cells from the lymph nodes, and enhanced tumor cell death [147,148]. Not many recently completed clinical trials have included BTK inhibitors in their regimen (Table 2), but newly designed clinical trials have begun to incorporate BTK inhibitors into their drug regimen (Table 3). Other BTK inhibitors are also being assessed *via in vitro* studies. Dasatinib, an inhibitor of tyrosine kinases, suppressed BCR-induced LYN, BCR-dependent EGR-1 upregulation, and cell survival in primary MCL cells. Similarly, PP2, a specific inhibitor of Src kinases suppressed constitutive LYN activation and induced apoptosis of MCL cells [149]. BTK is one of the major regulators of proliferation and cell survival in MCL and can be inhibited in the BCR pathway by ibrutinib, which down-regulates both phospho-STAT3 (pSTAT3) and NF-κB [150]. Ou et al. 2013 showed that microRNA-155 (miR-155) functions as a positive regulator of STAT3 while SOCS1 functions as a negative regulator of both STAT3 and NF-κB; they suggested that activity of ibrutinib and BCR inhibitors could be monitored *via* miR-155 [151]. In a recent *in vitro* study, ibrutinib and another SYK inhibitor, Fostamatinib, blocked chemotactic signaling by IL-1β, TNFα, and CCL5 which are required for MCL cell adhesion to human bone marrow stromal cells [148]. The combination of ibrutinib, spleen tyrosine kinase inhibitor R406, and HDAC inhibitor vorinostat increased apoptosis of MCL cells, which correlated with activation of caspase-3 and poly-(ADP-ribose) polymerase cleavage. Genomic profiling confirmed the down-regulation of NF-κB1/p105 and Cyclin D1 thus suggesting that this combination should be investigated in clinical studies [152]. Despite its promise, results have shown that MCL cell lines and patients can develop resistance to ibrutinib. Ibrutinib-resistant MCL cell lines (based on the therapeutic dose of 560 mg = 0.4 μg/μl) possessing a lack of normal BTK expression or presence of mutant BTK (C481S) were characterized by failure to inhibit phosphorylation of ERK and Akt signaling pathways, sustained PI3K-AKT activity, and activation of alternative NFκB pathway [153, 154].

### mTOR inhibitors

MCL pathogenesis involves PI3K/AKT/mTOR pathway activation. mTOR inhibitors, including temsirolimus (which is approved for relapse/refractory MCL in European countries), ridaforolimus, and everolimus impart their therapeutic benefit by inhibiting mTORC1 via allosterical binding. These agents have been tested for relapse/refractory MCL cases, but their efficacy in a single drug regimen has been low [155]. *In vitro* analysis indicated that low 4EBP1 expression and/or high eIF4E expression by lymphoma cells conferred resistance to mTOR inhibitors, explaining the poor therapeutic efficacy against MCL [156]. Several reports have suggested that treatment with NVP-BEZ235 could inhibit mTORC1 and mTORC2, as well as PKI3, and induce apoptosis of MCL cells by down-regulating constitutive Mcl-1 expression in chemo-naive MCL cell lines [157] and in bortezomib-resistant cell lines [158]. Other mTOR inhibitors such as WYE-132, AZD8055, PP242, and OSI-027 induced anti-proliferative activity and apoptosis *via* activation of the PUMA and BIM genes, and inhibition of the Akt signaling pathway [159]. Recent studies in an *in vivo* model with 13-197, a quinoxaline analog that specifically perturbs IκB kinase (IKK) β, a key regulator of the NF-κB pathway, disrupted both NF-κB and mTOR pathways resulting in downregulation of NF-κB and NF-κB-regulated genes such as Cyclin D1, Bcl-XL, and Mcl-1 as well as phosphorylation of S6K and 4E-BP1, the downstream mediators of the mTOR pathway [160]. None of the dual mTORC1/2 inhibitors have reached the clinical trial phase, but recently designed clinical trials have incorporated mTOR inhibitors in their treatment regimen for relapse/refractory MCL cases (Tables 2, 3).

### PI3K p110 inhibitors

PI3K functions early in the AKT signaling pathway. MCL is characterized by increased PI3K p110α expression especially in relapsed cases. The therapeutic efficacy of PI3Kp110 inhibitors such as CAL-101/idelalisib/GS-1101, SF1126/LY294002, rigosertib, and BYL719 have been validated *via in vitro* studies for hematological malignancies [161]. The oral agent idelalisib has been shown to block PI3K/AKT signaling and to promote apoptosis in MCL cell lines [162]. Idelalisib use in relapse/refractory cases of MCL yielded median PFS of 3.7 months and 1-year PFS of 22% [163]. A study by Chiron et al. 2013 [164] suggested that the tumoricidal effect of idelalisib is enhanced by G1-arrest of the cell cycle; therefore, idelalisib should be administered with concomitant inhibition of CDK4/CDK6 to increase the sensitivity of MCL cells to treatment regimen. The activity of idelalisib is largely non-overlapping with that of anti-CD20 antibodies (except for effects on phagocytic activity of microphages); thus, combination therapy with idelalisib and rituximab is found to be synergistic for patients with CLL [165]. In clinical trials, treatment with a pan-isoform PI3K and mTOR inhibitor SAR245409 (XL765) for relapse/refractory lymphoma produced partial remission [166]. These findings are being extended in additional clinical trials, one comparing idelalisib in combination with Rituximab *versus* BR for MCL (NCT01410513), and another trial employing idelalisib as single agent for relapse/refractory MCL (NCT01403636) (Table 3). In addition, PKC beta, a downstream regulator of BCR signaling has been found to be overexpressed in MCL. Enzastaurin, an oral serine/threonine kinase inhibitor, downregulates PKCbeta/PI3K/AKT pathways. It suppresses angiogenesis and proliferation resulting in induction of apoptosis. A phase 2 clinical trial has confirmed enzastaurin as a promising drug for treating relapse/refractory MCL [167]. *In vitro* studies demonstrated that treatment with enzastaurin and bortezomib together results in increased levels and an increased ratio of pro-apoptotic proteins (Bax, Bad, and Bim) relative to anti-apoptotic proteins (Bcl-2, Bcl-xL, and Mcl-1) [168]. Because of their ability to target two major pathological pathways thought to be involved in MCL, it would be reasonable to proceed with clinical trials aimed at incorporating these agents into the treatment regimen for MCL.

### AKT inhibitors and anti-tumor lipids

Activation of the AKT signaling pathway is known to cause proliferation by suppression of apoptosis as a consequence of direct phosphorylation of proapoptotic proteins such as Bad and pro-caspase-9. In preclinical studies, a combination of therapeutic agents that target the PI3K/AKT/mTOR signaling pathways has shown considerable anti-proliferative and pro-apoptotic activity against MCL cells. The efficacy of AKT inhibitors is usually monitored *via* expression levels of pAKT before and after treatment. Several AKT inhibitors are available including MK2206, perifosine, edelfosine, triciribine phosphate monohydrate (TCN-PM, VD-0002) and GSK2141795. MK2206 has been recently shown to significantly reduce phosphorylation of AKTser473 and to reduce phosphorylation of its target BADser110 in MCL cell lines. In addition, treatment with MK2206 also led to p21-coupled growth arrest in MCL cell lines [169]. An abnormal activation of lipid rafts by AKT has also been reported. Lipid rafts are cholesterol- and sphingolipid-rich membrane domains that act as platforms to colocalize proteins involved in intracellular signaling pathways. Lipid rafts control cell survival and cell death; they often become dysfunctional in cancers including MCL. The treatment with perifosine and edelfosine inhibits AKT and induces apoptosis in MCL cells and in xenograft-derived animal models; in the latter, pretreatment with PI3K inhibitor wortmannin potentiates the effect of perifosine [170].

### HSP90 inhibitors

Hsp90 (heat shock protein 90) is a molecular chaperon that facilitates correct protein folding; it stabilizes number of proteins that are involved in oncogenesis including NF-kappa B, BCR-ABL, NPM-ALK, AKT, and mutated p53 [171]. Thus, inhibitors of Hsp90 such as 17-AAG, suberoylanilide hydroxamic acid, 17-DMAG (17-dimethylaminoethylamino-17-demethoxygeldanamycin), GUT-70, and IPI-504 may have therapeutic value for treating various cancers. *In vitro* studies on MCL cell lines with 17-AAG led to downregulation of Cyclin D1, cdk4, and AKT, depletion of Bid, and activation of the intrinsic/mitochondrial caspase pathway [172]. The combination of 17-DMAG and HDAC inhibitor vorinostat exhibited a synergistic effect culminating in cell cycle arrest at G2/M and G1 and decreased expression of Cyclin D1, cdk4, c-Myc, c-RAF, and AKT leading to extensive apoptosis in MCL cell lines and primary cells [173]. Interestingly, GUT-70 demonstrated antiproliferative effects in forms of MCL in which p53 was mutated (mt-p53); it induced mitochondrial apoptosis with upregulation of NOXA and downregulation of Mcl-1 in mt-p53 cells, but affected only Mcl-1 expression in cells with wild type p53, indicating the specific activity of GUT-70 toward pathways affected by mutant p53 [174].

### Proteasome inhibitors

In addition to inhibiting the ubiquitin-proteasome complex, proteasome inhibitors induce the death response in transformed cells by inhibition of NF-κB-mediated anti-apoptotic response, upregulation of pro-apoptotic genes, and by stimulating accumulation of reactive oxygen species (ROSs). Several proteasome inhibitors have been tested against MCL. The first generation proteasome inhibitor bortezomib, a peptide boronic acid, targets all ubiquitin-tagged proteins, and acts *via* oxidative and endoplasmic reticulum (ER) stress leading to upregulation of proapoptotic protein NOXA as well as Bax and Bak [175]. Although bortezomib has been approved for treatment of relapse/refractory cases of MCL, a high incidence of peripheral neuropathy and gastrointestinal toxicity has limited its therapeutic usage [176]. Moreover, resistance to bortezomib has been reported among cases showing plasmacytic features and upregulation of IRF4 and CD38 [40]. Genome-wide DNA methylation analysis showed that bortezomib treatment resulted in hypomethylation of the NOXA gene; however, further *in vitro* studies demonstrated that administration of methyltransferase inhibitor decitabine (DAC) along with bortezomib overcame bortezomib resistance in relapsed/refractory MCL [177]. The combination of HSP90 antagonists with bortezomib has been found to be effective in nullifying bortezomib resistance mediated by ER chaperone BiP/Grp78 [178]. In addition, MLN9708, CEP-18770, and irreversible inhibitors such as carfilzomib and NPI-0052/Marizomib have shown promise in preclinical studies for MCL. Activities of these inhibitors can be monitored *via* enzymatic activity of each proteasomal subunit [179]. Carfilzomib, which is currently being evaluated in a phase 2 study for relapse/refractory MCL, induced apoptosis with the activation of JNK, Bcl-2, and mitochondria-related pathways. Its activity was dependent on the immunoproteasome subunit LMP2, which can function as a biomarker to monitor the therapeutic effect of carfilzomib [180]. While many proteasome inhibitors inhibit the catalytic activity of both constitutive proteasome and immunoproteasome, agents that can selectively inhibit the immunoproteasome have recently received attention. The immunoproteasome is a cytokine-inducible form of constitutive proteasome known to be expressed in lymphoid cell-derived hematological neoplasms. Immunoproteasome-specific inhibitors (IPSI) such as IPSI-001, PR-924, and PR-957 have not yet been assessed in clinical trials for MCL. Agents such as MLN4924 which inhibits the NEDD8 activating enzyme E1 leads to inhibition of the ligase complex, thereby limiting protein degradation by the proteasome. MLN4924 treatment has been shown to result in accumulation of NOXA protein in primary MCL and to induce apoptosis of MCL cells in a NOXA-dependent manner [181]. A phase 1 study for determining the therapeutic efficacy of MLN4924 has been completed. Similarly, inhibition of E3 ubiquitin ligase HDM2 by RG7112 leads to stabilization of selected proteins and suppression of the ubiquitin-proteasome system. RG7112 targets the murine double minute (mdm2) oncogene and activates p53 signaling in tumor cells [182].

### PARP inhibitors

Poly (ADP-ribose) polymerase (PARP) is a key component of DNA single strand break (SSB) repair machinery. Treatment with PARP inhibitors leads to conversion of unrepaired SSB lesions into DNA double strand breaks (DSBs) during DNA replication. Since MCL is characterized by inactivation of ATM gene and lack of DSB repair capacity, PARP inhibitors cause accumulation of extensive DNA DSBs and cell-death [183]. Although PARP inhibitors such as AG-014699/PF-01367338, AZD2281, ABT-888, XAV939, and others are mainly used for solid tumors, a few *in vitro* studies have demonstrated efficacy of PARP inhibitors against MCL. ATM-deficient MCL cell lines are especially sensitive to PARP inhibitor olaparib, the activity of which correlates with levels of ATM in p53-deficient gastric cancer. Cell lines deficient in both ATM and p53 genes were more sensitive than cells lacking ATM function alone [184]. A phase 1 clinical trial for MCL with CEP-9722 in combination with Gemcitabine and Cisplatin has recently been completed (NCT01345357) (Table 3).

### JAK/STAT inhibitors

JAK/STAT over-activation and mutations of SOCS1, an inhibitor of JAK/STAT pathway, have been reported in MCL [185, 186]. Degrasyn imparts antitumor activity in lymphoid tumors by inhibiting key growth and survival signaling (JAK/STAT) pathways. Degrasyn was shown to inhibit constitutively activated pSTAT3 and NF-κB. This results in inhibition of c-Myc, Cyclin D1, and Bcl-2 protein expression and upregulation of bax protein expression. Degrasyn has been found to potentiate the effect of Bortezomib against MCL cell lines [187]. There are other JAK inhibitors, such as AG490 are active against large cell lymphoma, but these have not been reported to be efficient against MCL cell lines [188].

### HDAC inhibitors

In cancer, it is common to find hypermethylation of tumor suppressor genes and hypomethylation of oncogenes. Histone deacetylases (HDACs) control gene expression by removing acetyl groups from histones and preventing the transcriptional machinery from obtaining access to their target gene. This results in transcriptional repression. HDAC inhibitors cause histone acetylation leading to restoration of expression of tumor suppressor and/or cell cycle regulatory genes in cancer cells resulting in suppression of proliferation of these cells [189]. HDAC inhibitors have shown promising anti-tumor activity (cell cycle arrest, cellular differentiation, and apoptosis) as well as inhibition of VEGF in both *in vitro* and *in vivo* models [190]. Many of the HDAC inhibitors are being tested against MCL. Among these, suberoylanilide hydroxamic acid (SAHA; vorinostat), an oral HDAC inhibitor, suppressed translation of Cyclin D1 mRNA by inhibiting the PI3K/AKT/mTOR/eIF4E-BP pathway [191]. The HDAC inhibitors valproic acid, MS-275 (a benzamide derivative), vorinostat, and panobinostat in combination with proteasome inhibitors (bortezomib, PR-171, NPI-0052, and others) have shown synergistic effects indicating the potential for further clinical studies [192, 193]. Similar effects have been achieved with a combination of HDAC inhibitors with other drug classes such as Bcl-2/BCL-XL antagonists [194], BCR signaling inhibitors [152], and mTOR inhibitors [195]. In a phase 2 clinical trial, PFS of 5.9 months was achieved with the HDAC inhibitor, vorinostat for relapse/refractory MCL (*n* = 9) however, vorinostat alone was not considered a promising agent [196]. In a phase 1 study with the HDAC inhibitor panobinostat and mTOR inhibitor everolimus a response was observed but at the stake of side effects especially significant thrombocytopenia [197] (Table 2).

### PIM kinase inhibitors

PIM kinases (proviral integration site for Moloney murine leukemia virus), which are proto-oncogenes, induce phosphorylation of critical downstream effectors of ABL (Abelson), JAK2 (janus kinase 2), and Flt-3 (FMS related tyrosine kinase) genes, and promote growth and survival through cell cycle regulation along with inhibition of apoptosis of malignant cells [198]. Overexpression of PIM kinases PIM1 and PIM2 has been reported in MCL [199–200]. PIM kinase inhibitors that target c-Myc-driven transcription and cap-dependent translation in MCL cells have shown promise. Some of the PIM kinase inhibitors are SGI-1776, SMI4a, LGB321, AZD1897, SEL24-B58, and AZD1208; among these SGI-1776 has shown high antitumor activity both *in vitro* and in an *in vivo* model of MCL [201, 202]. A combination of SGI-1776 and bendamustine imparted superior effects in inducing cell death and reducing levels of DNA, RNA, and protein synthesis as well as increasing DNA damage of MCL cells [203].

### BH3 mimetics/Bcl-2 family inhibitors

Bcl-2 is often overexpressed in MCL; hence, Bcl-2 is a potential target for MCL therapies. Several Bcl-2-selective small molecule BH3 mimetics function by binding to prosurvival members of Bcl-2 family and neutralizing their functional activity (sequestration of the proapoptotic Bcl-2 family members). This leads to release of pro-apoptotic family members from Bcl-2 and Bcl-xL, thereby inducing Bax- or Bak-dependent apoptosis [186]. Although many of these therapeutic agents have shown activities against MCL cells, their activity is determined by the activity/levels of Mcl-1, Bcl-1, and Bcl-xL [204]. ABT-737 and ABT-263 are capable of inducing rapid disruption of both Bcl-2/Bax and Bcl-2/Bik complexes in MCL cells and if these cells express the characteristic Bcl-2^high^/Mcl-1^low^ profile. Interestingly, addition of flavopiridol to ABT-737 nullified the Mcl-1-associated resistance of MCL cells to ABT-737. The authors argued that Bcl-2/Mcl-1 tumor cell profiles would indicate the benefit of ABT-737 therapy and should be incorporated to guide its use [205]. In another study, excessive activation of NF-kB pathway caused MCL cells with high Mcl-1 and Bxl levels to become resistant to Bcl-2 selective BH3 mimetic ABT-199. Addition of ibrutinib to ABT-199 was able to overcome the resistance since ibrutinib causes release of MCL cells from their microenvironment into the periphery [206]. GX15-070/Obatoclax, another BH3 mimetic, used alone or in combination with Bortezomib induced apoptosis in MCL cell lines *via* NOXA-mediated activation of Bak [207]. In clinical trials, this combination demonstrated overall response rate of 31% for relapse/refractory MCL cases [208] (Table 2).

### Novel antibodies and vaccines

Similar to rituximab, which targets CD20, other antibodies against CD antigens expressed by MCL cells have been tested. For example, a humanized anti-HLA-DR IgG4 mAb, IMMU-114 was reported to induce apoptosis in rituximab-resistant MCL cell lines *via* activation of ERK and JNK signaling [209]. Similarly, milatuzumab targets CD74, an integral membrane protein linked with promotion of B cell growth and survival. The combination of milatuzumab and rituximab induced rapid cell death, accompanied by generation of reactive oxygen species, loss of mitochondrial membrane potential, and potent inhibition of NF-κB pathway [210]. Otlertuzumab (TRU-016), an anti-CD37 protein, and polatuzumab vedotin, an antibody-drug conjugate containing an anti-CD79B monoclonal antibody conjugated to the microtubule-disrupting agent monomethyl auristatin E, showed acceptable safety and tolerability profiles on refractory/relapse MCL in a phase 1 study [211, 212]. Börschel et al., 2015 demonstrated that an antibody-cytokine fusion protein L19-IL2 that specifically delivers IL-2 to the tumor site by homing to the extra-domain B of fibronectin led to significant lymphoma growth retardation. The overall beneficial effect was potentiated by co-administration of rituximab [213]. Chen et al., 2015 have developed a novel vaccine based on targeting Cyclin D1 to Dendritic cells (DC) *via* the human DC surface receptor CD40 to elicit Cyclin D1-specific T cells, which is being tested in *in vivo* models [214]. Newer clinical trials have been designed to evaluate the clinical efficiency of some of these novel antibodies and vaccines (Table 3).

## CONCLUSIONS AND FUTURE DIRECTIONS

Over the last few decades, there has been growing improvement in the clinical outcome of mantle cell lymphoma. MCL is one of the most complex diseases, and involves aberrations in multiple signaling pathways, which lead to its poor prognosis. The contribution of the tumor microenvironment to the pathogenesis and outcome of MCL is equally complicated and, indeed, imparts modulatory effects on the outcome of this disease. The MCL International Prognostic Index (MIPI), which incorporates ECOG performance status, age, leukocyte count, and lactic dehydrogenase where inclusion of Ki67 proliferative index have been useful in stratification of high risk patients with probable poor prognosis. However, vigorous efforts are needed to identify target biomarkers which can be useful for predicting outcomes of MCL. It is important to personalize and individualize therapy for patients with poor prognosis. Combinations of traditional and novel therapeutic agents will be needed. Approaches will be needed that could minimize the recurrence/relapse rate for MCL and lead to improvement in the overall remission rate, PFS and OS. To date, *in vitro* studies have validated the beneficial effect of numerous drugs targeting various signaling pathways involved in MCL. In addition, phase 1 and phase 2 clinical trials with some of these drugs have been successful. Only a few have completed phase 3 trials; among these the combination of CDK inhibitors with agents targeting mTOR, BTK, and/or pan-isoform PI3K and mTOR inhibitors along with maintenance rituximab would appear to target MCL in a comprehensive manner. Although more clinical trials of various combinations of agents will definitely be needed in order to arrive at standardized therapies for untreated and refractory/relapse forms of MCL, the future looks promising.

## SUPPLEMENTARY TABLES






