# Supplementary material for: Mantle cell lymphoma in the era of precision medicine-diagnosis, biomarkers and therapeutic agents
Source: Oncotarget. 2016 Apr 23;7(30):48692–731. doi: 10.18632/oncotarget.8961 (PMC5217048; doi:10.18632/oncotarget.8961)
Supplement: Supplementary file 1 [file oncotarget-07-48692-s001.pdf]

**RNA/DNA Repair Regulator related markers**	MUM1/IRF4	Gene	Involved in DNA damage response, facilitates damage-induced chromatin changes	Up-Regulated	Involved in the DNA damage response pathway by contributing to the maintenance of chromatin architecture. Required for efficient DNA repair and cell survival following DNA damage.	Needs further research	IHC, PCR, whole-genome and/or whole-exome sequencing	216, 231
	WHSC1	Gene	DNA and Chromatin Binding	Up-Regulated	Lysine degradation	Needs further research	IHC, PCR, whole-genome and/or whole-exome sequencing	216, 233
	MLL2	Gene	Provides instructions for making lysine-specific methyltransferase 2D	Down-Regulated	Lysine degradation	Needs further research	IHC, PCR, whole-genome and/or whole-exome sequencing	216, 233
	MEF2B	Binding Factor	Interacts with Myo D family of transcriptional activators	Mutated	Immune response especially with T lymphocytes	Needs further research	IHC, PCR, whole-genome and/or whole-exome sequencing	216, 233
**Clusters of Differentiation**	CD56	Protein	Involved in neuron-neuron adhesion, outgrowth of neurites, neurite fasciculation	Usually negative but can be positive	Interferon, NCAM signaling for neurite out-growth, Cardiac Progenitor Differentiation	Ac2-26 inhibits CD56 function Heparin Sodium Salt inhibits CD56 function<br>RGDS peptide inhibits CD56	IHC, PCR, Flow Cytometry	216, 243
	CD200	Gene	Stimulates T-Cell proliferation	Negative	Class I MHC mediated antigen processing and presentation, Immuno-regulatory interactions between a Lymphoid and a non-Lymphoid cell	Not available	IHC, PCR, Flow Cytometry	216, 244
	CD5	Protein	Cell differentiation antigen expressed on thymocytes, T-cells, and B-cells.	Positive but can be negative	B Cell Receptor Signaling Pathway, Hematopoietic cell lineage	Not available	IHC, PCR, Flow Cytometry	7, 216
	CD43	Protein	Major glycoprotein of thymocytes and T-cells	Positive	Cell surface interactions at the vascular wall	Not available	IHC, PCR, Flow Cytometry	14, 15, 216
	CD23	Protein	Receptor for immunoglobulin E, essential for differentiation of B-cells	Usually negative but can be positive	Signaling by NOTCH2, GPCR, and IL4-mediated signaling events	Not available	IHC, PCR, Flow Cytometry	7, 14, 15, 216
	CD10	Protein	Cell membrane metallopeptidase widely distributed in	Positive	CD10 associates with p85, a PI3K subunit, and Lyn	Not available	IHC, PCR, Flow Cytometry	7, 14, 15, 216

			hematopoietic cells and their neoplasms		kinase indirectly prevents FAK activation by PI3K. Stimulates tumor suppressor PTEN leading to inhibition of Akt signaling pathway			
	CD103	Protein	Mediates adhesion of intra-epithelial T-lymphocytes to epithelial cell monolayers	Negative	ERK, Integrin and Actin Nucleation by ARP-WASP Complex	Not available	IHC, PCR, Flow Cytometry	7, 14, 15, 216
	CD20	Gene	Involved in B-cell activation and proliferation	Positive	Immune response Fc epsilon RI pathway, PI-3K cascade, Immune response NFAT during immune response	Ibritumomab, Rituximab and Tositumomab inhibit CD20	IHC, PCR, Flow Cytometry	7, 14, 15, 216
	CD49d	Gene	Makes up half of α4β1 lymphocyte homing receptor	Positive	ERK Signaling, Actin Nucleation by ARP-WASP Complex	natalizumab, a VLA-4 antibody inhibited adhesive interactions between MCL cells and MSCs in in viitro study	IHC, PCR, Flow Cytometry	216, 245
	CD79a/b	Gene	Required to initiate signal transduction cascade activated by binding of antigen to the B-cell antigen-receptor complex	Positive	B cell receptor signaling pathway,, Class I MHC mediated antigen processing and presentation, GPCR Pathway	Not available	IHC, PCR, Flow Cytometry	212, 216, 246
**Immune/Inflammatory Signaling related markers**	TLR (Toll like receptors)	Protein	Mediates and instigates altered immune response mainly implicated in malignant transformation, tumor progression and immune evasion in B cell malignancies	TLR1, TLR4, TLR7, TLR9 and TLR10-mRNA levels upregulated while TLR2, TLR3, TLR5 and TLR8 are found to be mutated/low	Toll-like receptor signaling via NF-Kappa B Pathway	Needs further research	IHC, PCR, ELISA, Quantibody Array, whole genome or whole exom sequencing	216, 247
	IL-12	Gene	Growth factor for T and NK cells, stimulates IFNGamma and IL10 production	Up-regulated	PEDF Induced Signaling, TGF-Beta, Toll-like receptor and Akt Signaling	IL-12 at 300 ng/kg starting at day 2 with Rituximab led to complete remission in 50% MCL cases	PCR, PCR, ELISA, Quantibody Array	69, 216
	IL-6	Protein	Pro-inflammatory cytokine, anti-inflammatory myokine, stimulates immune response	Up-Regulated	PEDF Induced Signaling, Apoptotic, GPCR , TGF-Beta and ERK Signaling	Tocilizumab/CNTO 328 (anti-IL6R monoclonal antibody) is available but has not been tried against MCL	PCR, PCR, ELISA, Quantibody Array	34, 69, 148, 216
	IL-2Rα	Gene	Constitutes high, medium, and low-affinity IL2A receptors	Up-Regulated	Apoptotic, PEDF TGF-Beta and Akt Signaling pathways	Treatment with mTORC1 inhibitor everolimus led to decrease in IL-2Ralpha in T cell lymphoma but has not been tested in MCL	PCR, PCR, ELISA, Quantibody Array	69, 216

	MIG (Monokine induced by Interferon-Gamma)	Protein	Affects growth, movement, and activation state of immune and inflammatory cells	Up-Regulated	PEDF, GPCR, ERK and Toll-like receptor signaling pathways	Combination of vorinostat and rituximab led to decrease in MIG levels in indolent NHL	PCR, PCR, ELISA, Quantibody Array	69, 70, 216
	IL-1RA	Protein	Inhibits pro-inflammatory cytokines, modulates interleukin-related immune responses	Up-Regulated	Interleukin-1, Interferon and NOD-like Receptor Signaling Pathways	Combination of vorinostat and rituximab led to decrease in MIG levels in indolent NHL	PCR, PCR, ELISA, Quantibody Array	69, 216
	IL-8	Protein	Chemotactic factor that attracts immune cells and involved in neutrophil activation	Up-Regulated	PEDF, TGF-Beta, Toll-like receptor , Peptide ligand-binding receptors and GPCR signaling pathways	Ibrutinib and Fostamatinib have been shown to decrease IL-8 levels	PCR, PCR, ELISA, Quantibody Array	69, 70, 148, 216
	IL-10	Protein	Cytokine synthesis inhibitory factor, stimulates proliferation and differentiation of T-cells	Up-Regulated	PEDF, TGF-Beta, Akt and ERK Signaling	Ibrutinib and Fostamatinib have been shown to decrease IL-10 levels	PCR, PCR, ELISA, Quantibody Array	148, 216
	IL-22	Protein	Contributes to in vivo inflammatory response	Up-Regulated	PEDF,TGF-Beta, Akt, ERK and PAK signaling pathways	NF-κB inhibitors, 6-amino-4-(phenoxyphenylethylamino)quinazoline (A and C) and E-2-fluoro-4′-methoxystilbene inhibit IL-22 expression in MCL cells	PCR, PCR, ELISA, Quantibody Array	216, 248
	LYN	Protein	Activates other kinases involved in activation and proliferation of immune cells	Up-Regulated	PI-3K cascade, Immune response, B Cell Receptor-antigen signaling pathways	Many Lyn peptide inhibitors are available; however, PP2 and dasatinib suppressed BCR-induced LYN and JNK phosphorylation as well as EGR-1 upregulation seen with MCL	PCR, PCR, ELISA, Quantibody Array	149, 216
	BTK	Gene	Plays role in B cell maturation and mast cell activation	Up-Regulated	PEDF, TGF-Beta, Immune response Fc epsilon RI pathway, Akt and ERK signaling pathway	Ibrutinib has shown promise against all B cell malignancies; other specific BTK inhibitors such as CC292, ACP-196 and ONO 4059 are in the pipeline	PCR, PCR, ELISA, Quantibody Array	125, 146, 216
**Signaling molecules**	NOTCH1	Gene	Play role in developmental processes through determining cell fate	Mutated	Pre-NOTCH Expression and Processing, Notch signaling pathway (KEGG)	Under clinical investigation	IHC, PCR, Whole transcriptome sequencing	22, 24, 216
	Mtorc1/2	Protein	Activates translation of proteins	Aberrant activation	Insulin receptor signaling cascade, PI-3K cascade, Signaling by FGFR and mTOR Pathways	Temsirolimus, everolimus and ridaforolimus have been shown to be active against MCL<br>Newer molecules such as NVP-BEZ235, WYE-132, AZD8055, PP242, and OSI-027 have been tested against MCL cell lines	IHC, PCR, WB	156, 216, 249
	WNT	Gene	Modulates cell behavior and fate during development	Mutated	Wnt signaling and signaling by GPCR	Needs further research	IHC, PCR, WB	216, 250
	AKT	Protein	Plays role in apoptosis, cell proliferation, and	Up-Regulated	PI-3K cascade, IL-9 and Apoptotic	Perifosine and MK2206 are under clinical invstigation	IHC, PCR, WB	169, 170, 216

			transcription		signaling pathways			
	SOCS1 (suppressor of cytokine signaling 1)	Protein	Suppresses cytokine signaling and JAK/STAT pathway	Mutated	Class I MHC mediated antigen processing and presentation mediated signaling, Interferon, IL-2 and IFN alpha/beta signaling pathways	Needs further research	IHC, PCR, WB	151, 185, 186, 216
	GSK3B	Protein	Inactivating agent of glycogen synthase	Up-Regulated	PI-3K cascade, FGFR, IL-9 and insulin Signaling Pathways. Involved with activation of cAMP-Dependent PKA	Needs further research	IHC, PCR, WB	216, 251
	JAK-2	Gene	Encodes for non-receptor tyrosine kinase JAK-2	Aberrantly activated	Signaling by Interleukins, Interferon and CNTF Signaling pathways	SB1518 has been shown o be active against MCL	IHC, PCR, WB	198, 216
**Chemokines**	CXCR4	Protein	Receptor for stromal-derived factor 1	Up-Regulated	Chemokine signaling pathway, GPCR, Akt and EphB-EphrinB signaling pathways	antibodies against CXCR4 and Plerixafor, a CXCR4 antagonist are active against MCL	IHC, PCR, Flow cytometery	32, 216, 245
	CXCR5	Protein	Receptor for CXCL13	Up-Regulated	Chemokine signaling pathway, GPCR, Akt and PEDF Induced Signaling	Needs further research	IHC, PCR, Flow cytometery	216, 245
**Cell Function/Maintenance**	HSP90	Protein	Assists other proteins to fold properly and stabilizes proteins against heat stress	Up-Regulated	Nucleotide-binding domain, leucine rich repeat containing receptor (NLR) signaling pathways, Fcgamma receptor (FCGR) dependent phagocytosis,	IPI-504 in combination with Bortezomib is effective agaisnt MCL. Other newer Hsp-90 inhibitors are being tested against MCL	IHC, PCR, FISH	174, 216
	SLC29A2 [solute carrier family 29 (equilibrative nucleoside transporter), member 2]	Gene	Transport of organic compounds and nutrients	Up-Regulated	ATP mediated transport of molecules via electrochemical gradient	Decynium 22, Dilazep dihydrochloride and Dipyridamole inhibit SLC29A2 function	IHC, PCR, FISH	216, 236

Supplementary Table 1: Table describing the key MCL biomarkers that inform diagnosis, prognosis, and functional drug interactions of MCL, along with their normal functions.
