# Supplementary material for: Mantle cell lymphoma in the era of precision medicine-diagnosis, biomarkers and therapeutic agents
Source: Oncotarget. 2016 Apr 23;7(30):48692–731. doi: 10.18632/oncotarget.8961 (PMC5217048; doi:10.18632/oncotarget.8961)
Supplement: Supplementary file 2 [file oncotarget-07-48692-s002.pdf]

Rituximab+ Bendamustine+ Ibrutinib	mechlorethamine + monoconal Ab+ BTK Inhibitor	untreated& relapsed/re-fractory NHL	lymphopenia, neutropenia , thrombocytopenia, and rash	48	1/1b	52%	N/A	N/A	72% {MCL ORR 94% (76% CR)}	N/A	2015	[96]
Temsirolimus +Bendamustine +Rituximab (BeRT)	(mTOR) inhibitor+ Alkylating agent+ monoconal Ab	r/r FL or MCL: Patients with 1-3 prior therapies	fatigue, nausea , mucositis, vomiting, Cough, diarrhea, pyrexia ,rash, leukopenia, neutropenia & thrombocytopenia	15	1/2	33.33 %	N/A	N/A	93% & for MCL:33%	19 months 67%	2015	[261]
R-DexaBEAM followed by HDT	Rituximab: monoclonal anti-CD20 antibody	relapsed or refractory B-cell lymphoma	all patients undergoing HDT experienced grade 4 haemato-toxicity	103	2	iNHL :32% aNHL :27%	N/A	N/A	aNHL: 62%, iNHL:78%	aNHL: 0.83Y(44% 7.3Y) iNHL: 3.7Y(72% 8Y)	2015	[262]
Bendamustine, lenalidomide and Rituximab	mechlorethamine+ immunomodulatory agent+ monoconal Ab	relapsed and refractory lymphomas	neutropenia was the most common grade 3 and 4 toxicity	20 14 evaluable	1	25%	10%	N/A	35%	N/A	2015	[263]
Temsirolimus and Bortezomib	(mTOR) inhibitor+ proteasome inhibitor	Relapsed or Refractory B-Cell NHL	no unexpected toxicities from the combination	39	2	7.70%	23%	N/A	31%	4.7 months	2015	[264]
Bortezomib and Alvocidib	reversible 26S proteasome inhibitor and flavonoid alkaloid CDK9 kinase inhibitor	multiple myeloma, indolent lymphoma, Waldenstrom macroglobulinemia, and mantle cell lymphoma	leukopenia, lymphopenia, neutropenia, and thrombocytopenia	44, assessable :39 (5 patients of MCL)	1	NA	NA	N/A	N/A	N/A	2014	[138]
Bortezomib-BEAM and autologous hematopoietic stem cell transplantation	Bortezomib: proteasome inhibitor	relapsed/refractory Diffuse and nodular forms of MCL	were manageable with no observed any treatment-related mortalities	42: 13 patients phase I & 29 patients phase II	I/II	At 1 year after ASCT 84% (P1) /81%(p2)	P1:3 % P2:4 %	P1:91% at 1Y & 67% at 5Y	P1:87 % /P2:89 %	p1:83 %/P2:83% (at 1 Y) and P1:32 % at 5Y	2014	[128]
Alisertib	Selective Aurora A Kinase Inhibitor	Relapsed and Refractory Aggressive B- and T-Cell NHL	grade 3/4 toxicities: neutropenia, leukopenia anemia, thrombo-cytopenia , stomatitis, febrile neutropenia &	48	2	10%	17%	N/A	27%	20% for 720 days	2014	[265]

			fatigue.									
Obatoclax and bortezomib	BH3 mimetic inhibitor+ proteasome inhibitor	relapsed/refractory MCL	Grade 3/4 events: thrombocytopenia, anemia and fatigue	13	I/II	23%	8%	NA	31%	N/A	2014	[208]
panobinostat + everolimus	histone deacetylase inhibitor+ mTOR inhibitor	Relapsed or Refractory Lymphoma	Grade 3/4 toxicity included thrombocytopenia , neutropenia , anemia , infection , fatigue & dyspnea	30, evaluable: 29	1	10%	23%	N/A	33%	N/A	2013	[197]
Lenalidomide + rituximab	L: immune-modulatory agent, R: anti-CD20 mAb	relapsed or refractory MCL	grade 3-4 haemato-logical toxicities	52 (phase1:14, phase1/2: 44)	1/2	phase 2:36 %	20%	24.3 months	57%	11.1 months	2012	[266]
Everolimus (RAD001)	mTOR inhibitor	Relapsed or Refractory MCL	criteria grade 3 including anemia thrombocytopenia and neutropenia	36, evaluable 35	2	5.70%	14.28 %	N/A	20%	5.5 months	2012	[267]
Bendamustine W/O Rituximab	Alkylating agent(mechlorethamine) W/O monoconal Ab	heavily pretreated NHL	neutropenia, anemia and thrombocytopenia, were primarily grade 0/1/2 in severity	175	multicenter retrospective study	29%	43%	10 months (70%)	71%	N/A	2012	[268]
Temsirolimus+rituximab	(mTOR) inhibitor +an antiCD20 antibody	relapsed or refractory MCL	Thrombo-cytopenia, neutropenia, fatigue, leucopenia, pneumonia, lymphopenia, pneumonitis & hyper-triglyceridae-mia	71 enrolled 69 assessable	2	19%	41%	N/A	59%	N/A	2011	[269]
Bendamustine+ Bortezomib+ rituximab	Bendamustine:Alkylating agent, Bortezomib: antineoplastic agent& Rituximab: anti-CD20 antibody	relapsed/refractory indolent and MCL NHL	nausea, neuropathy, fatigue, constipation and fever	30 evaluable: 29	2	51.70 %	N/A	N/A	83%	N/A	2010	[126]
RT-PEPC	rituximab: monoclonal anti-CD20 antibody + thalidomide: immune-modulatory agent plus prednisone: antiinflammatory; etoposide+procarbazine (both antineoplastic agents), and cyclophosphamide:	Recurrent MCL	grade 1/ 2 fatigue, rash, neuropathy, and cytopenias, thrombocytopenia and grade 3 and 4 neutropenia	25 enrolled, 22 evaluable	2	32%	41%	N/A	73%	10 months	2010	[270]

	alkylating agent											
Tipifarnib	Farnesyltransferase	relapsed/refractory MCL	no grade III–IV hematological toxicities	11	2	9%	0%	N/A	9%	3 months	2010	[271]
Yttrium-90-ibritumomab tiuxetan	Radio-immunotherapy	relapsed/ refractory MCL	primarily hematologic, Grade 3/4 thrombocytopenia& Grade 3/4 neutropenia	34	2	15.60 %	15.60 %	21 months	31%	6 months	2009	[272]
Gemcitabine, mitoxantrone, and Rituximab	Gemcitabine: Nucleoside Metabolic Inhibitor	relapsed or refractory MCL	grade 3–4 toxicities : neutropenia, leukopenia, thrombocytopenia& anemia	16	2	20%	27%	N/A	47%	N/A	2009	[273]
Bendamustine + Rituximab	Alkylating agent(mechlorethamine) + monoconal Ab	relapsed indolent B-cell and MCL non-Hodgkin's lymphoma	well tolerated; the primary toxicity was grade 3/4 neutropenia & grade 3/ 4 thrombocytopenia	66	2	41%	38%	N/A	92%	23 months	2008	[121]
R-HyperCVAD alternating with rituximab plus methotrexate-cytarabine	Chemo-immunotherapy	relapsed or refractory aggressive MCL	neutropenic fever , grade 3 or 4 neutropenia, and grade 3 or 4 thrombo-cytopenia	29	2	45%	48%	N/A	93%	N/A	2008	[274]
aza-epothilone Ixabepilone (BMS247550)	microtubule stabilization agent	relapsed or refractory indolent NHL & MCL	fatigue, myelosuppression, neuropathy & the most prominent advanced toxicities were leucopenia & lymphopenia	28<br>22 assessable	2	4.54%	22.70 %	8 months: 4.54 %	27%	N/A	2008	[275]
Bendamustine+ Mitoxantrone+ Rituximab (BMR)	B:Alkylating agent, M: chemotherapy R: monoconal Ab	stage III/IV relapsed or refractory indolent lymphomas and mantle cell lymphoma (MCL)	grade 3/4 toxicities	57	2	35%	54%	2 years	89%	19 months	2007	[132]
Enzastaurin	protein kinase C beta inhibitor (serine/threonine kinase inhibitor)	relapsed or refractory MCL	grade 3 anemia, diarrhea, dyspnea, vomiting, hypotension, and syncope	60	2	N/A	N/A	22 months	N/A	2 months	2007	[167]
Bortezomib	proteasome inhibitor	relapsed or refractory B-cell non-Hodgkin's	Grade 3/4 toxicities and three deaths.	60 (12of29 assessable MCL	2	A1:20 .5%	A2:2 0.5%	N/A	A1:41 % , A2:19	6 months (A1:42 %,	2005	[276]

		lymphoma.		(A1) & 4/2 1 assessable other BCL (A2))					%	A2:36 %)		

Supplementary Table 2: Table describing the details of the published clinical trials on the MCL drugs as single on in combination where A: arm , P: phase, Y: year, R-DexaBEAM: rituximab,examethasone, carmustine, etoposide, cytarabine and melphalan; HDT: high-dose therapy; aNHL: aggressive NHL; iNHL: indolent Lymphoma; N/A : Not available.
