# Supplementary material for: Mantle cell lymphoma in the era of precision medicine-diagnosis, biomarkers and therapeutic agents
Source: Oncotarget. 2016 Apr 23;7(30):48692–731. doi: 10.18632/oncotarget.8961 (PMC5217048; doi:10.18632/oncotarget.8961)
Supplement: Supplementary file 3 [file oncotarget-07-48692-s003.pdf]

14	**Bortezomib**<br><br>Velcade	MILLENNIUM PHARMS, 2003	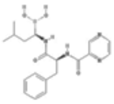	Antineoplastic Agent	Prescription/ intravenous injection or subcutaneous use	**Target:** proteasome subunit beta type-1 and type-5 inhibitor<br>**Mechanism of action:**<br>Inhibits the mammalian 26S proteasome which prevents targeted proteolysis, that can affect multiple signaling cascades within the cell. This disruption of normal homeostatic mechanisms leads to cell death.	**Hematologic:** thrombocytopenia, neutropenia, anemia.<br><br>**Other:** peripheral neuropathy.	[290]
15	**Rituximab**<br><br>Rituxan<br>Mabthera	GENENTECH, 1997<br>IDEC PHARMACEUTICALS CORP, 1997	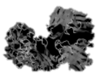	Antineoplastic [microtubule inhibitor], Monoclonal antibody	Prescription / Intravenous, Injection	**Target:** CD20<br>**Mechanism of action:**<br>Binds to the antigen CD20 and mediates B-cell lysis. Mechanisms of cell lysis include complement dependent cytotoxicity (CDC) and antibody dependent cell mediated cytotoxicity (ADCC).	**Other:** Tumor Lysis Syndrome, infections, renal toxicity, cardiac arrhythmias, bowel obstruction.	[291]
16	**Vorinostat**<br><br>Zolinza	MERCK, 2006	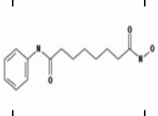	histone deacetylase inhibitor	Prescription / Oral	**Target:** histone deacetylase 1, 2, 3 and 6 (HDAC1, HDAC2, HDAC3, HDAC6) inhibitor<br>**Mechanism of action:**<br>1. Binds to the active site of histone deacetylases and act as a chelator for Zinc ions on the site.<br>2. Inhibition of histone deacetylases results in the accumulation of acetylated histones and acetylated proteins, including transcription factors crucial for the expression of genes needed to induce cell differentiation.	**Hematologic:** deep vein thrombosis, thrombocytopenia, anemia.<br><br>**Gastrointestinal:** nausea, vomiting, diarrhea, taste disorders.<br><br>**Other:** pulmonary embolism, hyperglycemia, chills.	[292]
17	**Alisertib**<br><br>MLN8237	Takeda Pharmaceutical Company Limited N/A<br>Millennium Pharmaceuticals, Inc. N/A<br>Selleck Chemicals, N/A	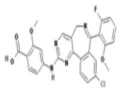	Aurora Kinase inhibitor	Prescription/ Oral	**Target:** aurora A kinase inhibitor<br>**Mechanism of action:**<br>It is selective and potent inhibitor of AAK. Aurora A directly binds to and regulates the turnover of N-myc, and is essential in N-myc amplified neuroblastomas. Also, Aurora A phosphorylate and regulate p53 function.	**Hematologic:** neutropenia, thrombocytopenia.<br><br>**Gastrointestinal:** diarrhea, nausea.<br><br>**Dermatologic:** alopecia.<br><br>**Other:** stomatitis, fatigue, somnolence.	[293]
**DRUGS UNDER TRIAL**								
17	**Idelalisib**<br><br>Zydelig	GILEAD SCIENCES INC, 2014	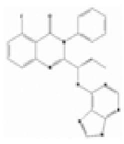	Antineoplastic agent,<br>Enzyme inhibitor	Prescription / Oral	**Target:** phosphatidylinositol 3 kinase inhibitor<br>**Mechanism of action:**<br>1. Promotes apoptosis in B-cell lines and primary cells from patients with different B-cell malignancies.<br>2. Inhibits constitutive and CD40-, TNF-α–, fibronectin-, and BCR-derived PI3K signaling leading to suppression of Akt activation.	**Gastrointestinal:** diarrhea, nausea, decreased appetite.<br><br>**Dermatologic:** rash.<br><br>**Other:** pyrexia, fatigue, upper respiratory infection and pneumonia.	[294]
18	**Everolimus**<br><br>Afinitor<br>Votubia<br>Zortress	NOVARTIS, 2009<br>N/A<br>NOVARTIS, 2010	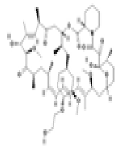	Kinase Inhibitor	Prescription / Oral	**Target:** mammalian target of rapamycin (mTOR) inhibitor<br>**Mechanism of action:**<br>Dual inhibition of the ER and PI3K/Akt/mTOR signaling pathways, providing synergistic inhibition of tumor.	**Gastrointestinal:** diarrhea, nausea, decreased appetite.<br><br>**Dermatologic:** rash.<br><br>**Other:** stomatitis, infections, fatigue, edema, abdominal pain, fever, asthenia, cough, headache.	[295]

19	**Ofatumumab**<br><br>Arzerra	GLAXO GRP LTD, 2009	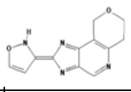	Antineoplastic agent, Monoclonal antibody	Prescription / Injection	**Target:** CD20<br>**Mechanism of action:**<br>1. Binds to both small and large extracellular loops of the CD20 .The CD20 molecule is not shed from cell surface and is not internalized following antibody binding.<br>2. Fab domain of ofatumumab binds to CD20 molecule & Fc domain mediates immune effector functions to result in B-cell lysis.	**Hematologic:** neutropenia, anemia.<br><br>**Gastrointestinal:** diarrhea, nausea.<br><br>**Dermatologic:** rash.<br><br>**Other:** pneumonia, pyrexia, cough, fatigue, dyspnea, bronchitis, upper respiratory tract infections.	[296]
20	**Panobinostat**<br><br>Farydak	NOVARTIS PHARMS CORP, 2015	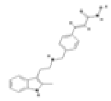	Antineoplastic, Histone Deacetylase Inhibitors	Prescription / Oral	**Target:** histone deacetylases (HDACs) inhibitor<br>**Mechanism of action:**<br>Inhibits multiple histone deacetylase enzymes that leads to apoptosis of malignant cells via multiple pathways like Notch signaling pathway	Constitutional symptoms:<br><br>**Hematologic:** myelosuppression.<br><br>**Gastrointestinal:** gastrointestinal symptoms.<br><br>**Other:** fatigue.	[297]
21	**Romidepsin**<br><br>Istodax	CELGENE, 2009	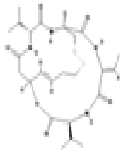	Antibiotics, Antineoplastic	Prescription / Infusion	**Target:** histone deacetylases (HDACs) inhibitor<br>**Mechanism of action:**<br>1.Inhibits removal of acetyl groups from lysine residues of N-terminal, maintaining transcriptionally active chromatin state.<br>2. Altered acetylation of nuclear and cytoplasmic proteins, although precise pathways by which it affects the cell cycle, apoptosis & angiogenesis have not been defined.	**Hematologic:** neutropenia, thrombocytopenia.	[298]
22	**Obatoclax**<br><br>GX15-070	PFIZER, N/A	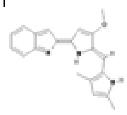	Immunomodulator, BCL-2 inhibitor, Antineoplastic	Under Trial/ Intravenous	**Target:** pan-BCL-2 inhibitor<br>**Mechanism of action:**<br>Disrupts survival of cancer cells by inhibiting the Bcl-2 pro-survival protein Mcl-1.	**Hematologic:** thrombocytopenia.<br><br>**Gastrointestinal:** diarrhea, nausea, constipation.<br><br>**Dermatologic:** rash.<br><br>**Other:** abdominal distension, abdominal pain, fatigue, weight loss, dehydration, neuropathy, somnolence, euphoric mood, cough	[299]
23	**Navitoclax**<br><br>923564-51-6<br>ABT 263<br>ABT-737<br>CS-0013	N/A	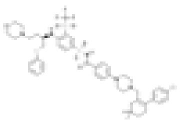	Antineoplastic, BCL-2 inhibitor	Under Trial/ Orally Bioavailable	**Target:** anti-apoptotic protein Bcl-2 inhibitor<br>**Mechanism of action:**<br>Antagonist of a subset of the B-cell leukemia 2 (Bcl-2) family of proteins with potential antineoplastic activity. Binds to apoptosis suppressor proteins Bcl-2, Bcl-XL, and Bcl-w, frequently overexpressed in a wide variety of cancers and are linked to tumor drug resistance. Inhibition of these apoptosis suppressors prevents their binding to the apoptotic effectors Bax and Bak proteins, thereby triggering apoptotic processes in cells overexpressing Bcl-2, Bcl-XL, and Bcl-w. This eventually reduces tumor cell proliferation.	**Hematologic:** thrombocytopenia.	[300]
24	**Dasiprotimut-T**<br><br>BiovaxID<br>Lympreva	Biovest International, Phase III Clinical Trials (US)	DasiprotimutT is an autologous lymphoma derived Ig idiotype-KLH [keyhole limpet haemocyanin] conjugate active immunotherapy product	Immunostimulant	Phase 3 Trial/ Vaccine	**Mechanism of action:**<br>Induces a tumour-specific cytotoxic T lymphocyte response	Nearly toxicity- free.	[301]

26	**Norcantharidin**<br><br>Aberela<br>Renova<br>Retin-A<br>Avita	Sigma-Aldrich, N/A	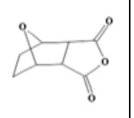	serine/threonine-protein phosphatase PP1-alpha catalytic subunit isoform 3	Pre clinical phase	**Target:**<br>1. Serine/threonine protein phosphatase 2A, catalytic subunit, alpha isoform.<br>2. Serine/threonine protein phosphatase PP1-alpha catalytic subunit.<br><br>**Mechanism of action:**<br>Inhibits serine/threonine protein phosphatase 2A and PP1-alpha. Causes apoptosis, inhibition of angiogenesis, and metastasis for many cell lines, and can affect multiple pathways controlling cell proliferation. Also, NCTD was found able to inhibit P-glycoprotein (P-gp) and overcome Multi drug resistance.	**Other:** Reduced cardiac and renal toxicity as compared to cantharidin.	[302]
27	**Puerarin**<br><br>Kakonein<br>Ciybio<br>Kudzu	Shannxi Ciyuan Biotech Ltd, N/A<br>Sigma-Aldrich, N/A<br>Thomson Pharma, N/A	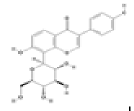	Isoflavone<br>Antiallergic<br>Antithrombotic	Pre clinical/ Supplement/ White powder(Available from china)	**Target**: The nuclear factor kappaB pathway.<br>**Mechanism of action:**<br>1. Inhibition of phosphorylation and degradation of inhibitor kappaB (I-kappaB), resulting in a reduction of p65NF-kappaB nuclear translocation.<br>2. Inhibits iNOS, COX-2 and CRP expression via suppression of NF-κB activation	Not available	[303]
28	**Atiprimod**<br><br>SK&F106615-A2<br>Azaspirane	Angene Chemical, N/A<br>Callisto Pharmaceuticals Inc, N/A	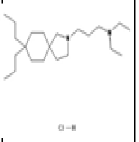	Anti-arthritic<br>Anti-inflammatory<br>Immunomodulator	Phase 2 trial /orally bioavailable	**Target:** JAK-STAT signaling pathway.<br>**Mechanism of action:**<br>Inhibits the phosphorylation of signal transducer and activator of transcription 3 (STAT3), blocking the signaling pathways of interleukin-6 and vascular endothelial growth factor (VEGF) and downregulating the anti-apoptotic proteins Bcl-2, Bcl-XL, and Mcl-1 inhibiting cell proliferation, inducing cell cycle arrest, and inducing apoptosis.	**Hematologic:** neutropenia.<br><br>**Gastrointestinal:** diarrhea, dyspepsia.<br><br>**Other:** liver enzyme elevation.	[304, 305]
29	**Dinaciclib**<br><br>SCH 727965	Merck &Co, N/A<br>Caymen chemicals, N/A<br>Selleck chemicals, N/A	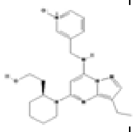	CDK inhibitor<br>Antineoplastic	Phase 2 trial /orally bioavailable	**Target:** p53 signaling pathway.<br>**Mechanism of action:**<br>Selectively inhibits cyclin dependent kinases CDK1, CDK2, CDK5, and CDK9; Inhibition of the unfolded protein response (UPR) through a CDK1 and CDK5-dependent mechanism. The inhibition of CDK1 and CDK2 may result in cell cycle repression and tumor cell apoptosis.	**Hematologic:** leukopenia, thrombocytopenia.<br><br>**Gastrointestinal:** gastrointestinal symptoms.<br><br>**Dermatologic:** alopecia.<br><br>**Other:** fatigue.	[306, 307]
30	**Alvocidib**<br><br>Flavopiridol	Sanofi-Aventis, N/A<br>Selleck Chemicals, N/A	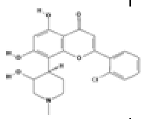	Flavonoid alkaloid<br>CDK9 kinase inhibitor	Phase 2 trial	**Target:** cyclin-dependent kinase (CDK) inhibitor<br>**Mechanism of action:**<br>1. Induces cell cycle arrest by preventing phosphorylation of cyclin-dependent kinases (CDKs) and by down-regulating cyclin D1 and D3 expression, resulting in G1 cell cycle arrest and apoptosis.<br>2. Inhibition of P-TEFb: suppressed transcription and decreased phosphorylation of RNA Pol II leading to a decrease in levels of proteins; cyclin D1, VEGF and Mcl-1.	**Hematologic:** granulocytopenia, anaemia.<br><br>**Gastrointestinal:** diarrhea.<br><br>**Other:** infection, fatigue.	[308, 309]

31	**Fostamatinib disodium**<br><br>Fostamatinib	Rigel Pharmaceuticals, N/A	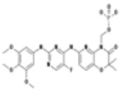	Spleen tyrosine kinase (Syk) inhibitors<br>Immunosuppressant<br>Antirheumatic<br>Antineoplastic	Phase3 trial/orally bioavailable	**Target:** NF-kappa B and B cell receptor signaling pathways.<br>**Mechanism of action:**<br>1. Inhibits Syk kinase-mediated IgG Fc gamma receptor signaling, resulting in inhibition of the activation of mast cells, macrophages, and B-cells and related inflammatory responses and tissue damage.<br>2. Syk kinase mediate diverse cellular responses, including proliferation, differentiation, and phagocytosis.	**Hematologic:** neutropenia, anemia.<br><br>**Gastrointestinal:** nausea, diarrhea.<br><br>**Other:** fatigue, increased AST, hypertension.	[310]
32	**Enzastaurin**<br><br>LY317615	Denovo Biopharma, N/A	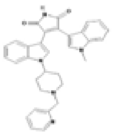	Antineoplastics / Indoles, Piperidines, Pyridines, Pyrrolidinones, serine threonine kinase inhibitor	Phase 3 Trial	**Target:** Protein kinase C beta (PKC-beta) inhibitor.<br>**Mechanism of action:**<br>Suppresses signaling through PKC-β and the phosphatidylinositol 3-kinase/AKT pathway to induce tumor cell apoptosis, reduce proliferation, and suppress tumor-induced angiogenesis.	**Hematologic:** anemia.<br><br>**Gastrointestinal:** diarrhea, vomiting.<br><br>**Other:** fatigue, dyspnea, hypotension, syncope.	[311]
33	**Palbociclib**<br><br>PD-0332991<br>Ibrance	Pfizer, 2015	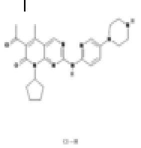	CDK4 and CDK6 inhibitor	Phase 3 Trial (Approved)/ Oral	**Target:** Cyclin-dependent kinase 4/6 (CDK4/6) inhibitor.<br>**Mechanism of action:**<br>CDK4, CDK6 and cyclin D1 regulate the G1- to S-phase cell-cycle transition via regulation of phosphorylation of the retinoblastoma (Rb) protein. Inhibition of these proteins leads to reduced phosphorylation of Rb, inhibition of downstream signalling, and increased tumor growth arrest.	**Hematologic:** leukopenia, anemia, thrombocytopenia.<br><br>**Gastrointestinal:** diarrhea, decreased appetite.<br><br>**Dermatologic:** alopecia.<br><br>**Other:** fatigue, upper respiratory tract infection, stomatitis, peripheral neuropathy, asthenia, epistaxis.	[312]
34	**Perifosine**<br><br>KRX-0401	Keryx Biopharmaceuticals/ Aeterna Zentaris, N/A	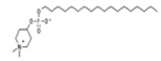	PI3K inhibitor, Akt inhibitor	Phase 3 Trial	**Target:** Protein kinase Akt (protein kinase B)<br>**Mechanism of action:**<br>1. Modulates membrane permeability, membrane lipid composition, phospholipid metabolism, and mitogenic signal transduction, resulting in cell differentiation and inhibition of cell growth.<br>2. Inhibits the anti-apoptotic mitogen-activated protein kinase (MAPK) pathway and modulates the balance between the MAPK and pro-apoptotic stress-activated protein kinase (SAPK/JNK) pathways inducing apoptosis.	**Gastrointestinal:** Moderate toxicity	[313, 314]
35	MK2206	Merck &Co, N/A	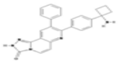	AKT Allosteric Inhibitor	Orally Bioavailable	**Target:** Protein kinase Akt (protein kinase B)<br>**Mechanism of action:**<br>Inhibits phosphorylation of Thr308 and Ser473 of Akt. Suppresses Akt signalling pathway and promotes cancer cell death as a single agent as well as in combination with other chemotherapeutic agents.	**Gastrointestinal:** nausea, vomiting, diarrhea.<br><br>**Dermatologic:** rash.<br><br>**Other:** fatigue, hyperglycemia.	[315]
36	**Uprosertib**<br><br>GSK2141795	GlaxoSmithKline, N/A	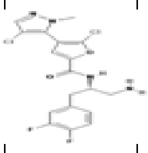	AKT Inhibitor, Antineoplastc	Phase 2 trial/orally bioavailable	**Target:** Protein kinase Akt (protein kinase B)<br>**Mechanism of action:**<br>It binds to and inhibits Akt activity. Inhibition of the PI3K/Akt signaling pathway, tumor cell proliferation and induction of tumor cell apoptosis.	**Dermatologic:** rash.<br><br>**Other:** hyperglycemia.	[316]

37	**SF1126/LY294002**<br>SF1126	Semafore Pharmaceuticals, N/A<br>SignalRx Pharmaceuticals, N/A	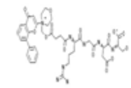	pan-PI3K inhibitor designed by conjugating RGD peptide to LY294002, Antineoplastics, Drug conjugates	Phase1 Trial	**Target:** PI3K and mTOR. DNA protein kinase, PIM1, and PLK1.<br>**Mechanism of action:**<br>1. Shuts down PI3K-Akt-mTOR signaling.<br>2. Decreases proliferation and has antiangiogenic and pro-apoptotic effect.	**Hematologic:** anaemia.<br>**Gastrointestinal:** nausea, diarrhoea.<br>**Dermatologic:** pruritus.<br>**Other:** fatigue, pyrexia, chills, anorexia, headache.	[317, 318]
38	**Tanespimycin**<br>17-AAG	Bristol-Myers Squibb, N/A	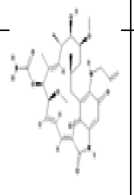	Hsp90 inhibitor	Phase 3 Trial	**Target:** HSP90<br>**Mechanism of action:**<br>Interferes with ATP-dependent chaperones activity, and degrades the client proteins by the proteasome.	**Hematologic:** anaemia.<br>**Gastrointestinal:** diarrhoea, vomiting.<br>**Other:** fatigue, muscle pain, transaminases elevation.	[319, 320]
39	**Ganetespib**<br>STA-9090	Synta Pharmaceutical, N/A	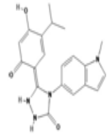	HSP90 inhibitor	Phase 3 Trial	**Target:** HSP90<br>**Mechanism of action:**<br>Competitive binding with the N-domain ATP-binding pocket of HSP90 disrupting the chaperone cycle. Degrades the client proteins by the proteasome.	**Gastrointestinal:** diarrhoea, nausea, decreased appetite.<br>**Other:** fatigue, insomnia.	[321]
40	**Pevonedistat**<br>MLN4924	Millennium Pharmaceuticals Inc, N/A	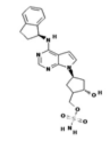	Small-molecule Inhibitor Nedd8 Activating Enzyme (NAE)	Phase1 Trial	**Target:** NEDD activating enzyme (NAE)<br>**Mechanism of action:**<br>Inhibits NAE resulting in inhibition of CRL neddylation and an increase in levels of CRL substrate proteins, causing induction of DNA replication by blocking degradation of Cdt-1. Novel approach to inhibit NF- B.	**Hematologic:** neutropenia, thrombocytopenia.<br>**Gastrointestinal:** nausea, diarrhea.	[322]
41	SNX-5422<br>SNX-2112.	Pfizer, N/A	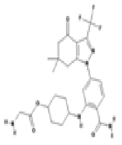	HSP90 inhibitor	Phase1 Trial	**Target:** HSP90<br>**Mechanism of action:**<br>Disruption of Hsp90 function has been shown to cause degradation of multiple Hsp90 client proteins, leading to inhibition of several key signaling pathways. This in turn results in inhibition of cellular proliferation.	**Gastrointestinal:** nausea, emesis, diarrhea.<br>**Other:** fatigue.	[323]
42	**Oprozomib**<br>PR047	Onyx Pharmaceuticals, N/A	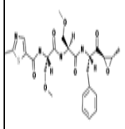	proteasome inhibitor	Phase 1 trial/ Oral	**Target:** Proteasome subunit beta type-5<br>**Mechanism of action:**<br>Inhibits the activity of the proteasome, this may result in an accumulation of unwanted or misfolded proteins. Disruption of various cell signaling pathways may follow, eventually leading to the induction of apoptosis and inhibition of tumor growth.	**Hematologic:** anemia, neutropenia.<br>**Gastrointestinal:** diarrhea, nausea.<br>**Dermatologic:** rash.	[324]

43	**Marizomib**<br><br>NPI-0052, Salinosporamide A	Nereus Pharmaceuticals Inc, N/A	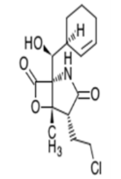	proteasome inhibitor	Phase 1 trial/ IV	**Target:** proteasome subunit beta type-1, 2 and 5<br>**Mechanism of action:**<br>Marizomib binds and inhibits the 20S catalytic core subunit of the proteasome resulting in an accumulation of poly-ubiquitinated proteins that causes disruption of cellular processes, cell cycle arrest, the induction of apoptosis, and the inhibition of tumor growth and angiogenesis.	**Gastrointestinal:** nausea, vomiting.<br><br>**Other:** fatigue, headache, dizziness, fever.	[325]
44	**Cerdulatinib**<br><br>PRT062070	Portola Pharmaceuticals Inc, N/A	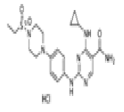	Syk-JAK inhibitor,antineoplastic, anti inflammatory	Phase 1 trial/ Oral	**Target:** Syk, JAK1, and JAK3<br>**Mechanism of action:**<br>Binds to and inhibits the activity of Syk, JAK1, and JAK3 with preferential inhibition of JAK1 and JAK3-dependent cytokine-mediated signaling and functional responses.	**Hematologic:** anemia, neutropenia, hematochezia.<br><br>**Other:** fatigue, hypotension, increased AST.	[326]
45	**Sorafenib tosylate**<br><br>Nexavar	BAYER HLTHCARE, 2005	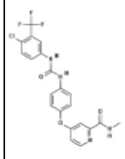	Multi-kinase inhibitor	Prescription/ Oral	**Target:** Simultaneous targeting of the Raf/Mek/Erk pathway.<br>**Mechanism of action:**<br>Sorafenib interacts with multiple intracellular (CRAF, BRAF and mutant BRAF) and cell surface kinases (KIT, FLT-3, VEGFR-2, VEGFR-3, and PDGFR-ß). Several of these kinases are thought to be involved in angiogenesis, thus sorafenib reduces blood flow to the tumor. Sorafenib is unique in targeting the Raf/Mek/Erk pathway. By inhibiting these kinases, genetic transcription involving cell proliferation and angiogenesis is inhibited.<br>Interferes with BCR signaling, protein translation and modulates the microenvironment prosurvival signals in MCL.	**Hematologic:** thrombocytopenia, neutropenia.<br><br>**Gastrointestinal:** diarrhea.<br><br>**Dermatologic:** hand/foot reactions.	[327]
46	Piceatannol	N/A	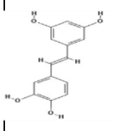	protein-tyrosine kinase	Phase 1 trial	**Target:** Different protein tyrosine kinases<br>**Mechanism of action:**<br>Inhibits the purified thymocyte protein-tyrosine kinase, p40 and competes for peptide or protein substrate binding site. It also inhibits the activity of the p56lck protein-tyrosine kinase. Inhibits protein<br>tyrosine kinase Syk and induces apoptosis.	N/A	[328]
47	**Belinostat**<br><br>PXD-101<br>Beleodaq	Spectrum pharmaceutical , 2014	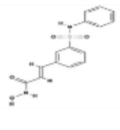	HDAC inhibitor, antineoplastic	Phase 2 trial/IV, infusion, oral	**Target:** HDACs<br>**Mechanism of action:**<br>Targets HDAC enzymes, inhibiting tumor cell proliferation, inducing apoptosis, promoting cellular differentiation, and inhibiting angiogenesis. This agent may sensitize drug-resistant tumor cells to other antineoplastic agents, possibly through a mechanism involving the down-regulation of thymidylate synthase.	**Hematologic:** neutropenia, thrombocytopenia.<br><br>**Gastrointestinal:** adynamic ileus.<br><br>**Dermatologic:** pruritis, rash/erythema, edema.	[329]

48	**Entinostat**<br><br>MS-275	Syndax, N/A<br>Merck, N/A	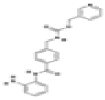	HDAC inhibitor, antineoplastic	Phase 3 trial	**Target:** HDAC1 and 3<br>**Mechanism of action:**<br>Entinostat binds to and inhibits histone deacetylase. This agent appears to exert dose-dependent effects in human leukemia cells including cyclin-dependent kinase inhibitor 1A (p21/CIP1/WAF1)-dependent growth arrest and differentiation at low drug concentrations; a marked induction of reactive oxygen species (ROS); mitochondrial damage; caspase activation; and, at higher concentrations, apoptosis.	**Gastrointestinal:** diarrhea.<br><br>**Dermatologic:** rash.<br><br>**Other:** muscular weakness, fatigue.	[330]
49	**Veliparib**<br><br>ABT-888	AbbVie, N/A	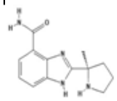	PARP inhibitor	Phase 2 trial/ Oral	**Target:** NF-kappa B signaling pathway.<br>**Mechanism of action:**<br>A poly(ADP-ribose) polymerase (PARP) -1 and -2 inhibitor. It inhibits PARPs, inhibiting DNA repair and potentiating the cytotoxicity of DNA-damaging agents. Hinders efficient DNA repair and survival of proliferating cells exposed to mild genotoxic stresses.	**Hematologic:** leukopenia, neutropenia, anemia.<br><br>**Gastrointestinal:** nausea, vomiting.<br><br>**Other:** dehydration, elevated alanine transaminase level, fatigue.	331
50	**Pacritinib**<br><br>SB1518	Baxter, N/A<br>Cell therapeutics, N/A	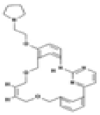	Janus kinase inhibitor	Phase 2/oral	**Target:** JAK-STAT signaling pathway<br>**Mechanism of action:**<br>Competes with JAK2 for ATP binding, it might result in inhibition of JAK2 activation, inhibition of the JAK-STAT signaling pathway, and so caspase-dependent apoptosis.	**Hematologic:** anemia, thrombocytopenia.<br><br>**Gastrointestinal:** diarrhea, nausea, vomiting, constipation, abdominal pain, edema peripheral, abdominal distension.<br><br>**Other:** fatigue, cough, dizziness, insomnia, anorexia.	332
51	**Carfilzomib**<br><br>PR171<br>Kyprolis	Onyx pharmaceutical, 2012	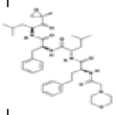	Proteasome inhibitor	Prescription/ IV	**Target:**Proteasome subunit beta type-5.<br>**Mechanism of action:**<br>Inhibition of the proteasome leads to a decreased cellular proliferation, ultimately resulting in cell cycle arrest and apoptosis of cancerous cells.<br>Induces apoptosis of MCL cells mediated by the activation of JNK, Bcl-2, and mitochondria-related pathways. In addition, carfilzomib inhibits the growth and survival signaling pathways NF-κB and STAT3.	**Hematologic:** thrombocythemia, anaemia, thrombocytopenia, lymphopenia.<br><br>**Gastrointestinal:** nausea.<br><br>**Other:** fatigue, hypophosphatemia, hyponatremia.	333

Supplementary Table 4: The pharmacological details of the drugs currently in use or being developed for MCL
